# MiR-24 Is Required for Hematopoietic Differentiation of Mouse Embryonic Stem Cells

**DOI:** 10.1371/journal.pgen.1004959

**Published:** 2015-01-29

**Authors:** Lynn Roy, Emmanuel Bikorimana, Danica Lapid, Hyewon Choi, Tan Nguyen, Richard Dahl

**Affiliations:** 1 Harper Cancer Research Institute, South Bend, Indiana, United States of America; 2 Microbiology and Immunology, Indiana University School of Medicine, South Bend, Indiana, United States of America; 3 Dept. of Biological Sciences, University of Notre Dame, Notre Dame, Indiana, United States of America; St Jude Children’s Research Hospital, United States of America

## Abstract

Overexpression of miRNA, miR-24, in mouse hematopoietic progenitors increases monocytic/ granulocytic differentiation and inhibits B cell development. To determine if endogenous miR-24 is required for hematopoiesis, we antagonized miR-24 in mouse embryonic stem cells (ESCs) and performed *in vitro* differentiations. Suppression of miR-24 resulted in an inability to produce blood and hematopoietic progenitors (HPCs) from ESCs. The phenotype is not a general defect in mesoderm production since we observe production of nascent mesoderm as well as mesoderm derived cardiac muscle and endothelial cells. Results from blast colony forming cell (BL-CFC) assays demonstrate that miR-24 is not required for generation of the hemangioblast, the mesoderm progenitor that gives rise to blood and endothelial cells. However, expression of the transcription factors Runx1 and Scl is greatly reduced, suggesting an impaired ability of the hemangioblast to differentiate. Lastly, we observed that known miR-24 target, Trib3, is upregulated in the miR-24 antagonized embryoid bodies (EBs). Overexpression of Trib3 alone in ESCs was able to decrease HPC production, though not as great as seen with miR-24 knockdown. These results demonstrate an essential role for miR-24 in the hematopoietic differentiation of ESCs. Although many miRNAs have been implicated in regulation of hematopoiesis, this is the first miRNA observed to be required for the specification of mammalian blood progenitors from early mesoderm.

## Author Summary

Studies of mouse embryos and embryonic stem cells (ESCs) have defined the ontogeny of mammalian embryonic hematopoietic cells. The ESC differentiation system has been valuable for dissecting the molecular regulation of the development of mesoderm into HPCs. Extracellular signals regulate a complex network of transcription factors to direct embryonic hematopoietic development. Mammalian miRNAs have previously not been described to regulate this genetic network during embryonic hematopoiesis. However, a role for miRNAs in producing the hemangioblast, and hemogenic endothelium in Xenopus has been described. Our work with ESCs demonstrates a specific requirement for the miRNA, miR-24, in the development of hematopoietic progenitors cells (HPCs). Antagonizing miR-24 in ESCs does not affect generation of BL-CFCs, the *in vitro* equivalent of the hemangioblast, but does compromise the ability of those BL-CFCs to produced HPCs. Expression of transcription factors required for HPC production downstream of the hemangioblast, Scl, and Runx1, is greatly reduced by antagonizing miR-24. These results identify miR-24, as a mammalian miRNA required for the development of blood from newly formed mesoderm.

## Introduction

MicroRNAs (miRNAs) are small (~22 nucleotide) RNA molecules that regulate gene expression post-transcriptionally. They are implicated in important cellular processes such as apoptosis, proliferation, and differentiation [1]. Work from many laboratories, including our own, shows that miRNAs regulate hematopoietic progenitor cell fate decisions and immune cell function [2–4]. However, the role of miRNAs in regulating the earliest hematopoietic stem and progenitor cell development is less characterized. Additionally, a role for miRNAs has not been described for directing the development of the mammalian hemangioblast or hemogenic endothelium, the early mesoderm that gives rise to primitive and definitive hematopoietic cells [5, 6].

Studies of mouse embryos and embryonic stem cells (ESCs) have defined the ontogeny of mammalian embryonic hematopoietic cells [7]. During embryogenesis primitive hematopoietic progenitor cells (HPCs) are produced first in the yolk sac, and then in the embryo proper. Definitive hematopoiesis begins in the aorta-gonad-mesenephros (AGM) region off the embryo, and later switches to the fetal liver. These tissues arise from a subset of mesoderm, the lateral plate mesoderm. In vertebrates, the initial hematopoietic and endothelial lineages are generated simultaneously in the same region of the embryo [8, 9]. This suggests that these lineages arise from a common mesodermal derived progenitor termed the hemangioblast. The first direct evidence demonstrating the existence of the hypothesized hemangioblast came from work with ESCs. Keller and colleagues identified a progenitor from ESC derived embryoid bodies (EBs) that formed a blast colony in methylcellulose, which contained clonal blood and endothelial cells [10]. They termed this progenitor the blast colony-forming cell (BL-CFC), which was proposed to be the in vitro equivalent of the hemangioblast. These progenitors were enriched in the EB population of cells that coexpresses the mesoderm specific transcription factor T (Brachyury), and the tyrosine kinase receptor Flk1 [11]. This same group later demonstrated the transient existence of BL-CFCs in the gastrulating mouse embryo within the primitive streak [12]. In the aortic-gonad-mesenephros (AGM) region of the mouse embryo, blood has been shown to arise from differentiated endothelial cells termed the hemogenic endothelium, which appears independent of a hemangioblast cell [13, 14]. During differentiation of ES derived BL-CFCs, it has also been observed that BL-CFC cells form a hemogenic endothelium intermediate during the development of HPCs [5].

The ESC differentiation system has been valuable for dissecting the molecular regulation of the development of mesoderm into HPCs. Extracellular signals including BMPs, FGFs, VEGF, Notch ligands, and Wnts [15–18] regulate a complex network of transcription factors to direct embryonic hematopoietic development. The Ets transcription factor Etv2 is essentially for hemangioblast specification in zebrafish [19, 20]. A direct connection to mammalian hemangioblast development has not been shown, but Etv2 is necessary for development of embryonic blood progenitors and vasculature as demonstrated by mouse gene targeting experiments [21]. Etv2 expression is correlated with markers of hemangioblast and hemogenic endothelium in differentiating ESCs, and genetically acts upstream of the transcription factor Scl in blood development [22, 23]. Scl is required for the differentiation of BL-CFC cells into hemogenic endothelium [5, 24]. After formation of hemogenic endothelium, another transcription factor, Runx1, is required for the production of definitive HPCs [5, 25]. These signaling pathways, and transcription factors are known to regulate and/or be regulated by miRNAs, so it would be surprising if miRNAs did not have a role in the production of HPCs for mesoderm [26–29]. Additionally, global knockdown of miRNAs in Xenopus embryos results in an ablation of hemangioblast development [30].

We recently identified miR-24 as a miRNA that regulates the development of adult hematopoietic progenitor cells [3]. MiR-24 is produced from two distinct mammalian genes *mirn23a*, and *mirn23b*. The *mirn23a* gene codes for three miRNAs, miR-23a, miR-27a, and miR-24–2, which is expressed as a pri-miRNA from its own independent transcription unit. *Mirn23b* codes for miRs-23b, -27b, and 24–1, and is embedded in an intron of the aminopeptidase O (*AMPO*) gene. The mature miR-24 miRNAs (miRs-24–1, -24–2) produced from both clusters are identical in sequence whereas the mature miRs-23a/b and-27a/b differ by one nucleotide at the 3 prime ends (outside of the seed sequence which is responsible for target selection). The *mirn23a* miRNAs are enriched in hematopoietic cells compared to *mirn23b* miRNAs [3]. When we retrovirally expressed the miRs-23a, 27a, and 24 in hematopoietic progenitors we observed that these miRNAs inhibited B cell development and potentiated the development of monocytes and granulocytes [3]. Additionally, we observed that miR-24 is necessary and sufficient to generate this phenotype.

To determine if miR-24 is required for proper monocyte and granulocyte development, we antagonized miR-24 in murine embryonic stem cells (ESCs) by infecting them with a lentivirus encoding an shRNA targeting miR-24. Stable knockdown clones were differentiated into embryoid bodies (EBs). To our surprise, hematopoiesis was dramatically inhibited in miR-24 knockdown (KD) EBs. In this report we examine the requirement for miR-24 in blood development from ESCs. In addition, we interrogated Trib3 as a critical target for miR-24 to repress during the development of HPCs. Previously; Trib3 was identified as a direct miR-24 target in vascular smooth muscle cells [31]. Trib3 modulates BMP/ Smad signaling through inhibition of the E3 ubiquitin ligase Smurf1 [32], that negatively regulates Smad1 and Smad 5. MiR-24 targeting of Trib3 potentially links it to known pathways regulating embryonic hematopoiesis [33–36].

## Results

### MiR-24 is required for blood formation from embryonic stem cells

**Fig 1:**
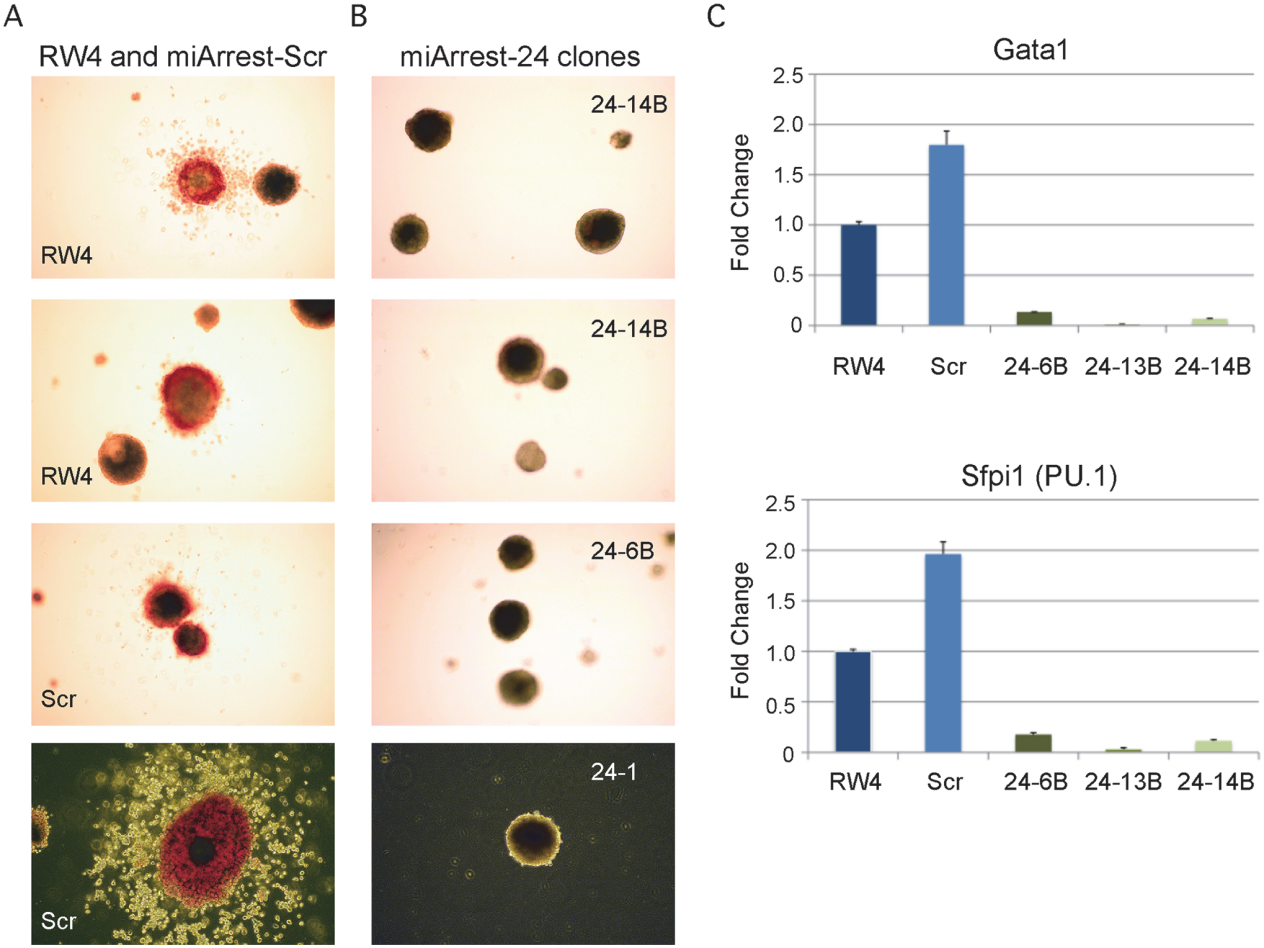
MiR-24 is required for blood development from mouse embryonic stem cells. ESC clones were generated from RW4 cells infected with either miArrest-24 (ShRNA targeting miR-24) or miArrest-SCR (Scrambled shRNA) lentivirus. **A)** RW4 wildtype, SCR (miArrest-SCR), and **B)** miArrest-24 clones (24–1, 24–6B, and 24–14B) were differentiated into EBs for 14d in methylcellulose media. Top 3 panels are 25X magnification, and bottom panels are 50X. **C)** Quantitative RT-PCR analysis of expression of the hematopoietic transcription factors Gata1, and Sfpi1 from RNA obtained from the indicated ESC clones differentiated into EBs in liquid culture for 6d. The decrease in expression observed in the miArrest-24 clones compared to uninfected and miArrest-SCR infected RW4 clones was significant for Gata1, and Sfpi1 with respective P values of <0.0005, and <0.0008 as determined by unpaired t-test. Error bars represent the standard error of the mean (SEM).

Previously, we demonstrated that enforced expression of miR-24 in hematopoietic progenitors promoted myeloid (monocytes and granulocytes) development [3]. To determine if miR-24 is required for the development of myeloid cells, we infected mouse ESCs with a lentivirus expressing an shRNA that binds and inhibits miR-24 (miArrest-24, Genecopoeia, Rockville, MD). For controls, we generated ESCs expressing a scrambled non-targeting shRNA. The miR-24 shRNA clones had reduced expression of miR-24 that persisted for at least 6 days of EB differentiation as determined by quantitative reverse transcriptase PCR (Q-RT-PCR) (S1A, B Fig.). Decreased expression of miR-24 may underestimate the reduction in miR-24 as we may detect miR-24 bound inactive to the shRNA. MiR-24 knockdown did not affect the expression of clustered miRNAs, miRs-23a and 27a (S1C Fig.). When ESC lines were differentiated, we observed that, in contrast to wildtype and scrambled control shRNA expressing ESCs (Fig. 1A); the miR-24 knockdown clones did not generate EBs with hemoglobinized cells (Fig. 1B). This suggested that blood was not being made and this was confirmed by a dramatic reduction in the expression of hematopoietic transcription factors Gata1 (P<0.0005) and Sfpi1 (P<0.0008) in miR-24 KD d6 EBs compared to wildtype and scrambled control derived EBs (Fig. 1C). We also performed hematopoietic colony assays with cells isolated from d9 EBs as previously described [37]. On average we observed less than 1 hematopoietic colony generated from 50 miR-24 knockdown EBs (S2 Fig.). To support the conclusion that the hematopoietic defect is due specifically to knockdown of miR-24, we also generated miR-24 knockdown ESCs with an independent shRNA expressing lentiviral vector (miRZIP, Systems Biosciences, Mountain View, CA). A similar defect in blood production in EBs was observed using this alternative method of targeting miR-24 (S3A, S3B Figs.).

### MiR-24 is required for the generation of hematopoietic progenitor cells

**Fig 2:**
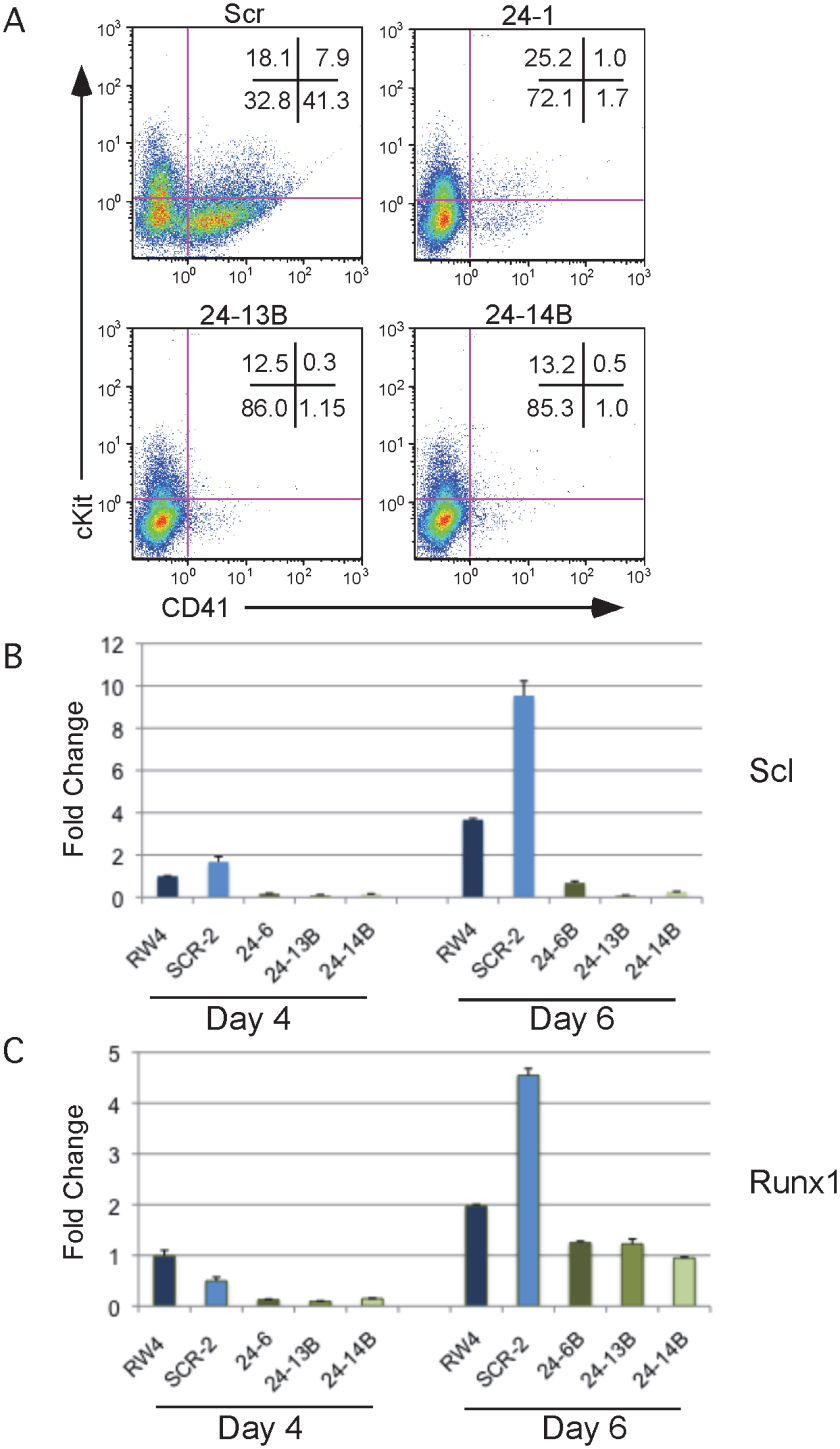
MiR-24 is required for HPC generation. ESC clones infected with control miArrest-SCR, or miArrest-24 lentivirus were differentiated into EBs for 6d in liquid culture and analyzed by flow cytometry and quantitative RT-PCR. **A)** Flow cytometry analysis of CD41 and cKit (CD117) cell surface expression on single cells isolated from EBs generated from the indicated ESC clones. CD41+cKit- population contains primitive HPCs and CD41+cKit+ contains primitive and definitive HPCs. **B, C)** Expression of the transcription factors **B)** Scl, and **C)** Runx1, which are required for the development and/or function of hemogenic endothelium. RNA was isolated from d4 and d6 EBs. The decrease in expression observed in the miArrest-24 clones compared to uninfected and miArrest-SCR infected RW4 cells was significant at d4 for Scl, and Runx1 with respective P values of <0.007, and <0.002, as well as at d6 with respective P values of <0.003, and <0.008. P values were determined by unpaired t-test. Error bars represent the SEM.

We observed that miR-24 was necessary for blood development [3]. To determine if this requirement was early or late in embryonic hematopoiesis, we performed flow cytometry on cells derived from d6 EBs to determine if HPCs were being made in the absence of miR-24. EBs generate HPCs that can be identified by the cell surface expression of CD41 [38]. There were almost no HPCs derived from miR-24 KD clones (Figs. 2A, S4). A similar defect in HPC production was observed from ESCs with miR-24 targeted by the miRZIP lentiviral vector (S3C Fig.). Consistent with this result, we observed significantly decreased expression of the transcription factors Scl and Runx1 between wildtype/ scrambled controls and miR-24 KD clones in d4 EBs with respective P values of less than 0.007, and 0.002, as well as at d6 with P values of less than 0.003, and 0.008 (Fig. 2B, C). These two factors are expressed in HPCs and function downstream of the hemangioblast [24, 25].

### Absence of miR-24 does not result in a general differentiation defect

**Fig 3:**
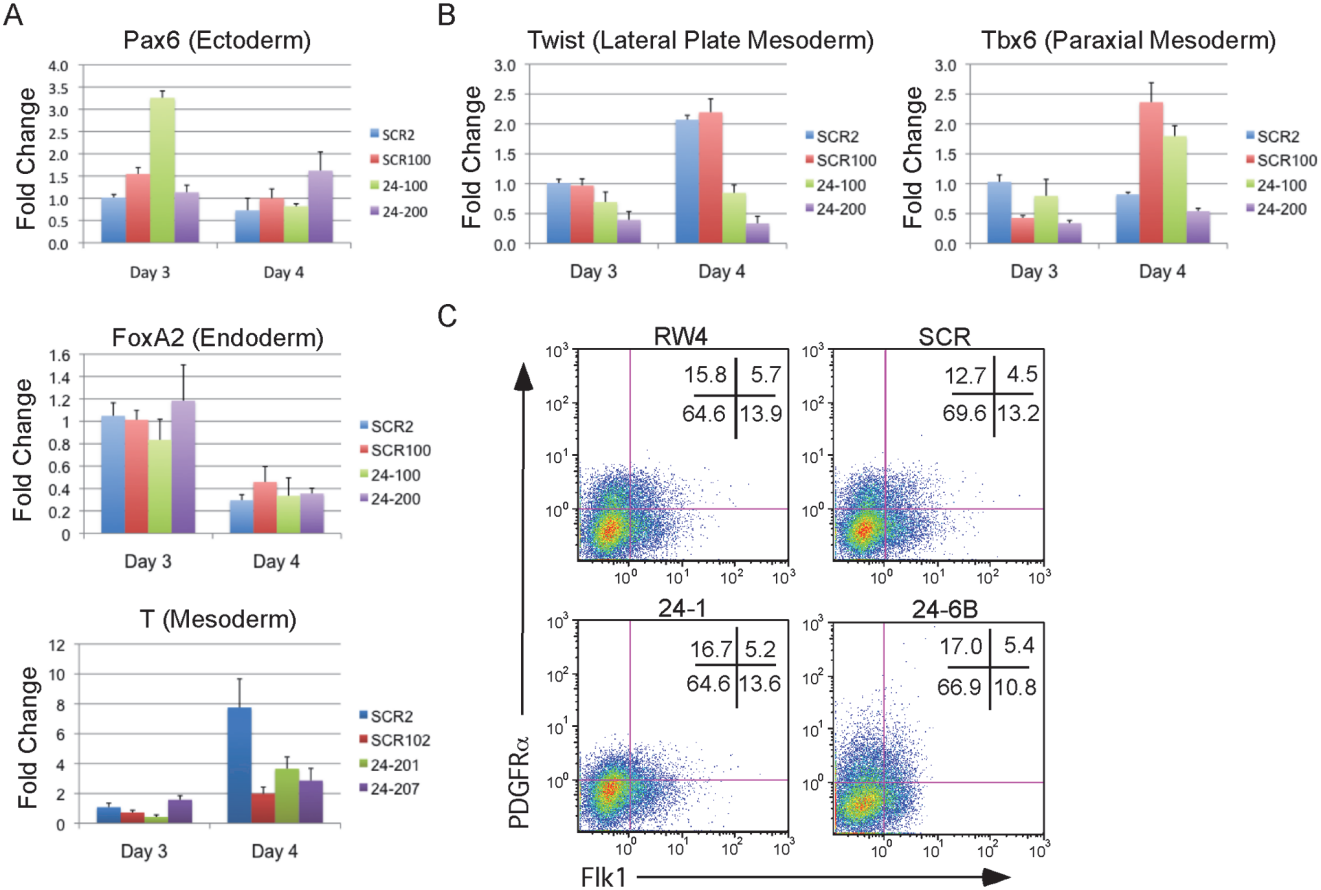
MiR-24 is not required for the differentiation of embryonic stem cells into embryoid bodies. Two independently derived miArrest-SCR, and miArrest-24 ESC clones were differentiated into EBs. RNA was collected at d3 and d4 of differentiation, and used to analyze gene expression by quantitative RT-PCR. **A)** Formation of the germ layers was analyzed by assaying expression of Pax6 (Ectoderm), FoxA2 (Endoderm), and T (Mesoderm). No significant differences were observed in gene expression comparing scrambled shRNA clones to miR-24 shRNA clones. **B)** To further analyze mesoderm differentiation the expression of lateral plate mesoderm gene Twist, and paraxial plate mesoderm gene Tbx6 were assayed. No significant difference was observed in Tbx6 expression, however the decrease in Twist expression was significant at d3 and d4 with respective P values of <0.002, and <10^-8^ respectively. Error bars in panels A and B represent the SEM. **C**. The indicated ESC clones were differentiated in liquid culture for 4d. Development of paraxial and lateral plate mesoderm was analyzed by flow cytometry. Lateral plate mesoderm is FLK+PDGFRα-, paraxial mesoderm is FLK1-PDGFRα+, and nascent uncommitted mesoderm is FLK1+PDGFRα+.

The defect in hematopoiesis could be due to a global defect in development. To examine if early germ layer tissue was being formed from miR-24 KD ESCs, 2 scrambled control, and 2 miR-24 KD clones were differentiated and RNA harvested at d3 and d4. Q-RT-PCR analyzed expression of Pax6 (Ectoderm), FoxA2 (Endoderm), and T (Brachyury, mesoderm). No significant differences (P>0.05) in the expression of these genes were observed when comparing the average values obtained from scrambled and miR-24 KD clones (Fig. 3A). Hematopoietic cells in the developing EB and embryos are derived from mesoderm tissue. Mesoderm gives rise to paraxial and lateral plate mesoderm with the later giving rise to hematopoietic cells. To determine if there was a defect in nascent mesoderm differentiation we analyzed expression of the genes Twist (lateral plate) and Tbx6 (paraxial). We did not observe a significant effect of miR-24 knockdown on Tbx6 expression, though expression was variable within control and 24 KD clones. MiR-24 antagonism did lead to a significant decrease in Twist expression at both d3 (P<0.002) and d4 (P< 10^-8^)(Fig. 3B). This suggested a reduction in lateral plate mesoderm, consistent with the observed decrease in hematopoiesis. To further investigate this, we performed flow cytometry analysis of d4 EBs to determine if early mesoderm populations were being made in miR-24 KD clones. Lateral plate mesoderm is identified by the cell surface expression of FLK1, and the lack of expression of PDGFRα. In contrast, paraxial mesoderm is identified by expression of PDGFRα and lack of expression of FLK1 [39, 40]. Nascent mesoderm coexpresses both FLK1, and PDGFRα, and can give rise to either lateral plate or paraxial mesoderm. By flow cytometry, we did not observe a defect in mesoderm production, as lateral plate (FLK1+PDGFRα-) and paraxial mesoderm (FLK1-PDGFRα+) were observed in miR-24 KD EBs (Fig. 3C).

**Fig 4:**
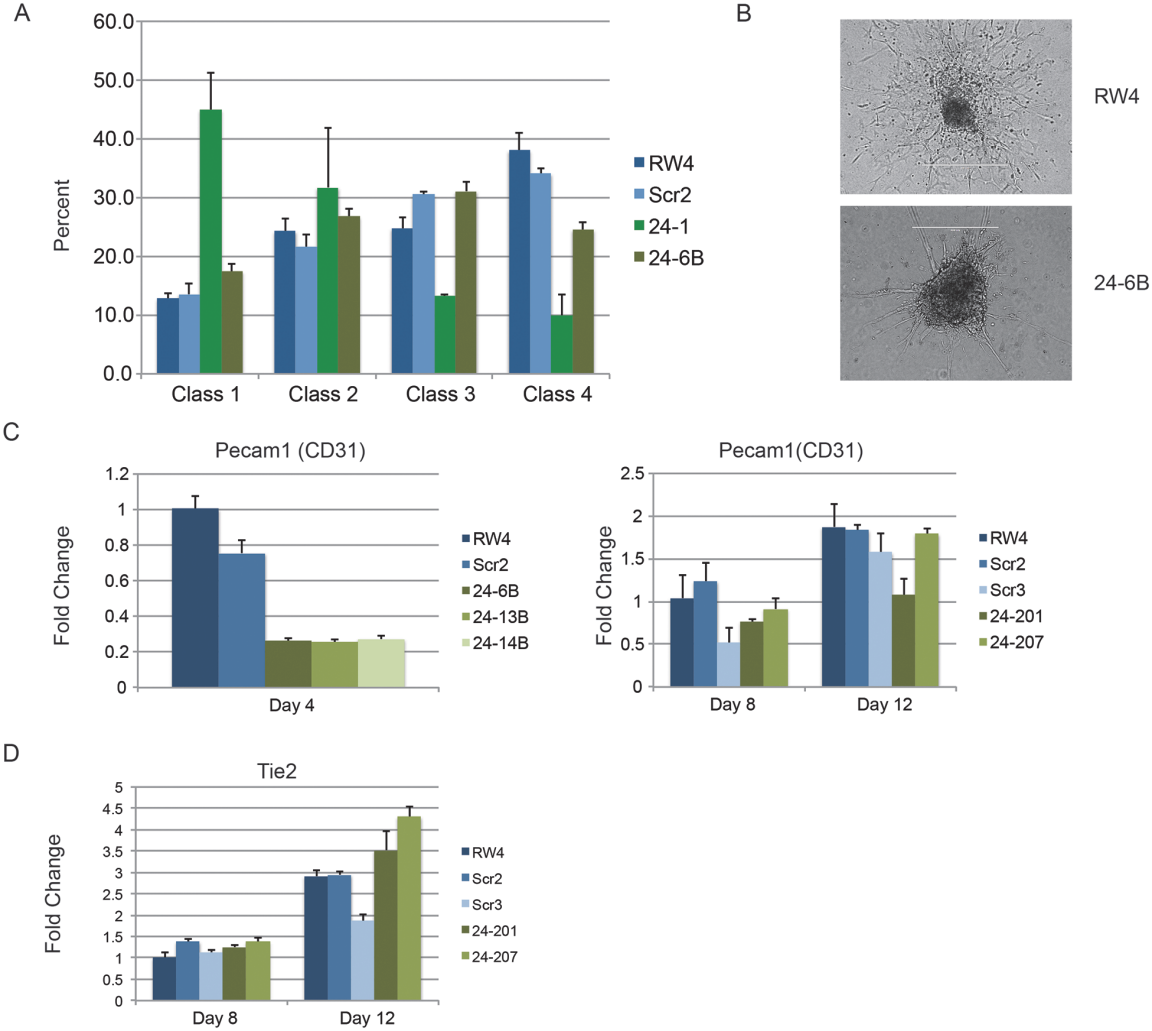
Endothelial cell development impaired in miR-24 knockdown embryoid bodies. **A)** D11 EBs plated in collagen with angiogenic growth factors for an additional 4d were scored according to 4 standard classes of vascular sprout formation: I- no sprout formation, II- few sprouts, III- many sprouts but no network, IV- many sprouts with network [37]. The decrease in class 4 sprouts observed in miR-24 knockdown clones, 24–1, and 24–6B compared to RW4, and SCR shRNA control clones is significant with a P value <0.002. **B)** Day 11 EBs differentiated additional 4 days in collagen media. Representative image of sprouting from control and miR-24 KD EBs. Bar denotes 400uM. **C)** Expression of endothelial gene Pecam1 (CD31) in RNA isolated from d4, d8, and d12 EBs. The decrease in expression observed at d4 in the miArrest-24 clones compared to uninfected and miArrest-SCR infected RW4 was significant for Pecam1 with a P value of <0.0002 as determined by unpaired t-test. Significant differences were not observed at d8 or d12. **D)** Expression of endothelial associated gene Tie2 was analyzed at d8 and d12 of EB development. No differences in Tie2 expression were observed at d8, however there was a slight increase in Tie2 expression at d12 with P<0.0007 as determined by unpaired t-test. Error bars represent the SEM.

Besides blood, lateral plate mesoderm is responsible for the production of cardiac muscle and blood vessels (endothelial cells). To examine production of blood vessels we performed vascular sprout assays [41]. Forty d6 miR24 KD EBs and control-scrambled shRNA EBs were plated onto collagen-coated plates in media containing angiogenic cytokines. Sprout formation was assessed 6 days later (total 12 days of culture). EBs were scored in blinded fashion according to 4 standard classes based on vascular sprout formation: I- no sprout formation, II- few sprouts, III- many sprouts but no network, IV- many sprouts with network [41]. In the development of class 3 and 4 sprouts, we saw large variability between the two miR-24 KD clones examined (Fig. 4A). However there was a slight decrease in the generation of class 4 sprouts between miR-24 KD and control ESC clones, which was statistically significant (P<0.002). The sprouts on individual EBs appeared morphological similar between control and MiR-24 KD EBs (Fig. 4B). We also analyzed expression of Pecam1 (CD31) to evaluate blood vessel/ endothelial cell development. Pecam1 expression was evaluated in d4, d8, and d12 EBs. Initially Pecam1 expression was decreased between miR-24 KD and control EBs at d4 (P<0.0002) (Fig. 4C). However by d8 and d12, there are no longer any significant differences in Pecam1 expression (P>0.05). We also examined the expression of endothelial gene Tie2 at d8 and d12 of EB differentiation. There was no significant difference in Tie2 at d8, however there was a small increase in Tie2 detected in the miR-24KD clones at d12 (P<0.0007) (Fig. 4D).

**Fig 5:**
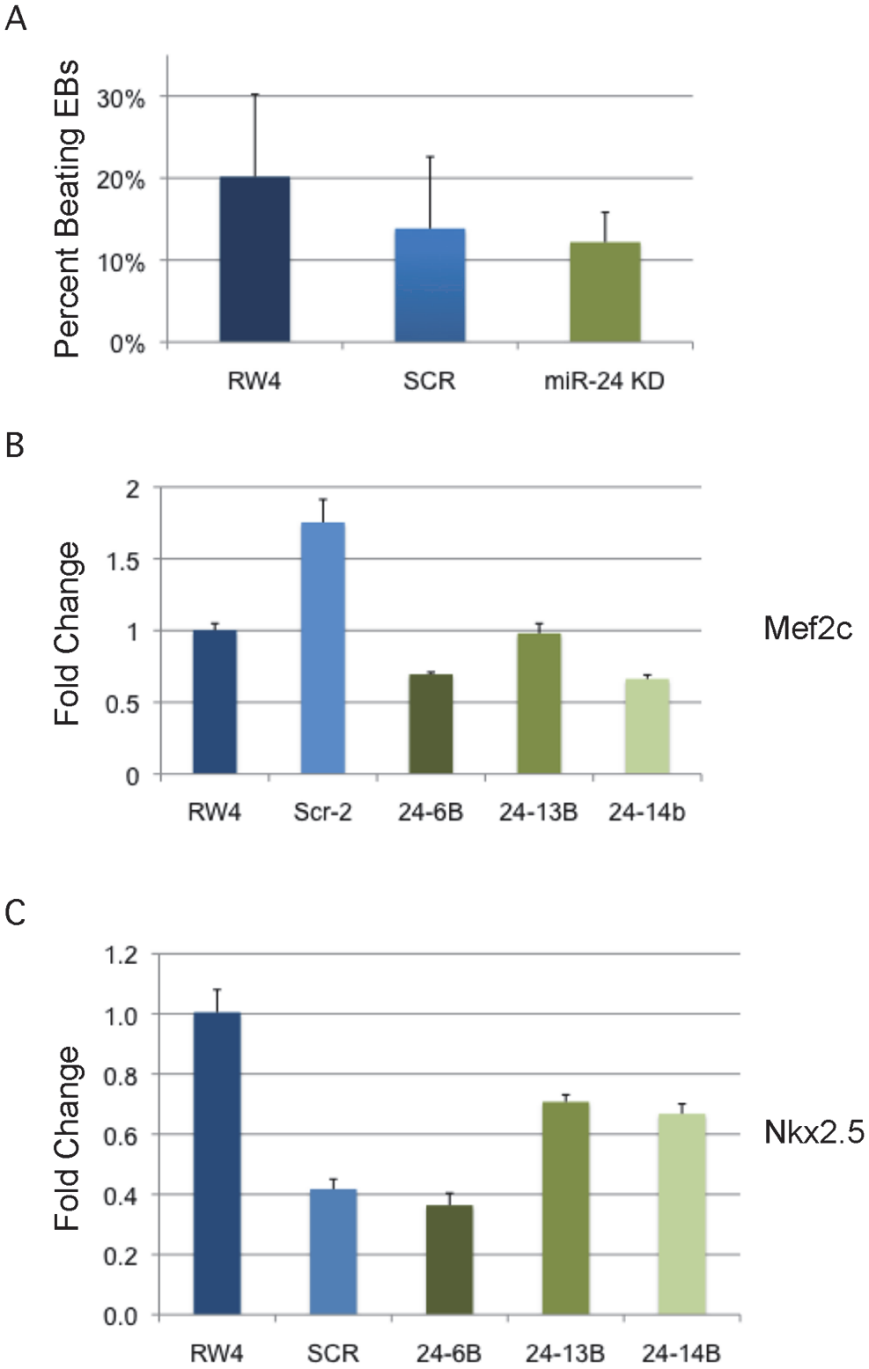
MiR-24 is not required in EBs for cardiac muscle development. **A)** RW4 parental cells, miArrest-Scr ESC clones, and miArrest-24 ESC clones were differentiated into EBs and by hanging drop and individual EBs were transferred to 96 well plate. After 5 days the wells were examined for the presence of beating cells indicative of the presence of cardiac muscle. For each individual differentiation the percent wells positive for beating cells was recorded. RW4 cells n = 6, miArrest-Scr n = 8, and miArrest-24 n = 16. For miArrest-Scr, 2 independent clones were examined, and for miArrest-24, 6 independent clones were examined. **B, C)** Quantitative RT-PCR analysis of expression of the cardiac muscle transcription factors **B)** Mef2c, and **C)** Nkx2.5. For both Mef2c, and Nkx2.5 the differences in expression observed in the miArrest-24 clones compared to uninfected and miArrest-SCR infected clones was *NOT* considered significant with P values >0.05 as determined by unpaired t-test. Error bars represent the SEM.

To assay cardiac development, we generated EBs by hanging drop and then replated individual EBs into single wells of a 96 well plate. After 5d cardiac muscle differentiation was evaluated by determining the percentage of wells positive for contracting cells. We did not observe a significant difference in the frequency of wells positive for beating EBs in the miR-24 KD cultures compared to control cultures (Fig. 5A). We also assayed expression of cardiac muscle genes Nkx2.5, and Mef2c between control EBs (wildtype and Scrambled) and miR-24 KD clones. Our analysis did not demonstrate any significant differences in the expression of these two genes (Fig. 5B,C). Mef2c expression does appear to be decreased if we just compare the miR-24 KD clones to the scrambled control clone.

### Perturbation of miR-24 does not effect generation of blast colony-forming cells

**Fig 6:**
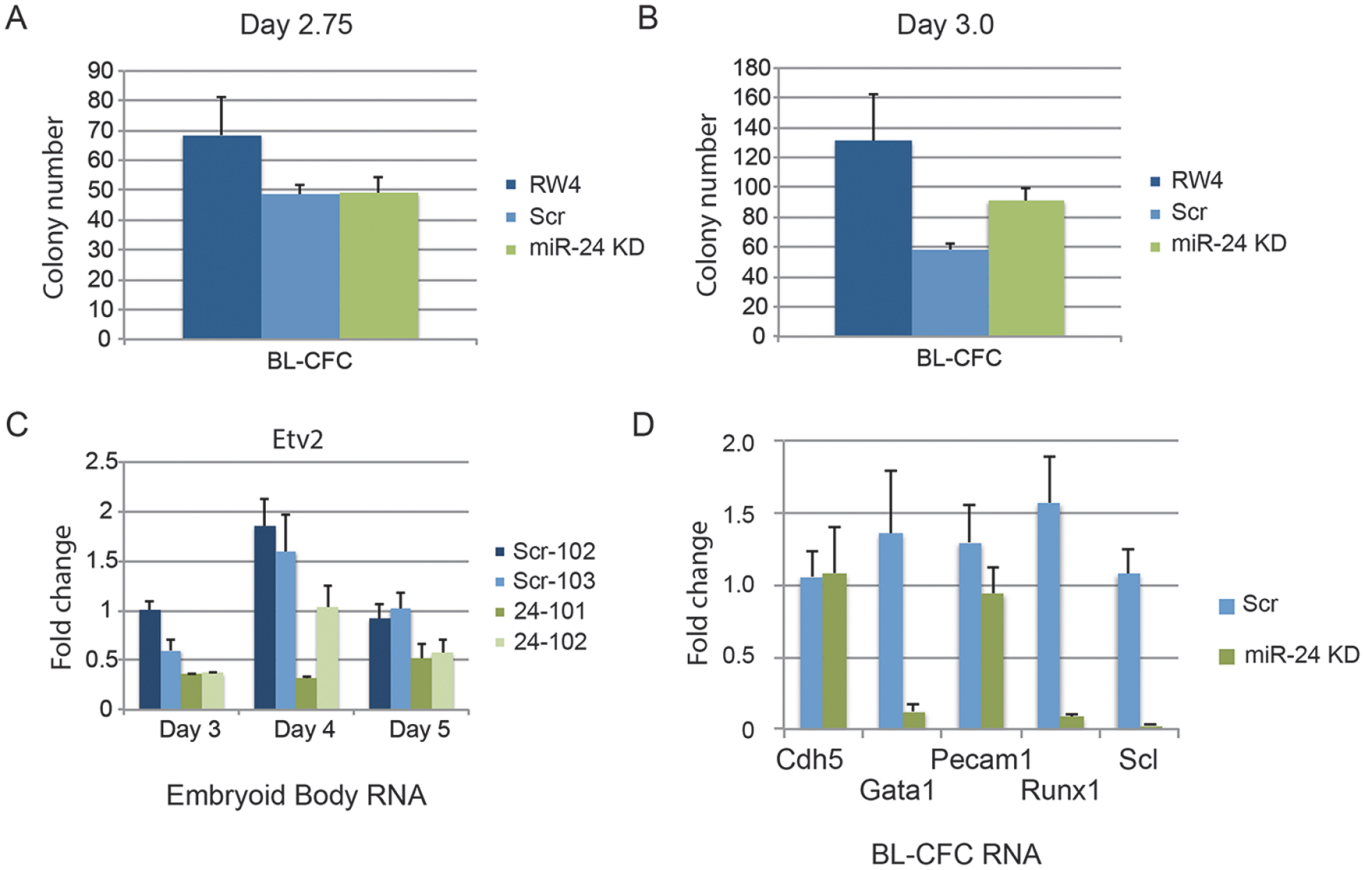
BL-CFCs develop from EBs with antagonized miR-24. RW4 parental cells, miArrest-Scr clones, and miArrest-24 clones were differentiated in liquid culture for **A)** 2.75d or **B)** 3.0d. EBs were dissociated into single cell suspensions and plated in methylcellulose for growth of BL-CFCs. 4d later BL-CFCs were counted. 2 independent miArrest-Scr clones were examined for d2.75 and d3 differentiations. 4 miArrest-24 clones for d2.75 and 7 miArrest-24 clones for d3 were examined. For d2.75, RW4 n = 4, miArrest Scr n = 6, and miArrest-24 n = 8. For d3.0, RW4 n = 8, miArrest Scr n = 8, and miArrest-24 n = 16. **C)** RNA was isolated from d3, d4, and d5 EBs derived from the indicated ESC clones. Expression of the transcription factor Etv2 was examined. The decrease in expression observed in the miArrest-24 clones compared to uninfected and miArrest-SCR infected RW4 cells was significant at d3 (P<0.006), d4 (P<0.002), and d5 (P<0.006) as determined with by unpaired t-test. **D)** Twenty BL-CFC colonies from individual cultures were isolated and used to generate cDNA. Quantitative-RT-PCR was performed with the indicated gene specific primers. Data was averaged from results obtained from the differentiation of 3 scrambled clones, and 3 mir-24 KD clones. The decreases in Gata1, Runx1, and Scl expression are significant with respective P values of P<0.001, P< 10^-4^, and P<10^-7^.

Since there was not a general defect in mesoderm production, we further investigated when in HPC development miR-24 is required. Although lateral pate mesoderm is diminished as evaluated by Twist expression, it does develop as observed by flow cytometry (FLK1/PDGFRα) and assaying cardiac and vascular sprout development. We performed BL-CFC assays with parental RW4 cells, scrambled shRNA clones and miR-24 shRNA clones. ESCs were differentiated into EBs for either 2.75d or 3.0d, and dissociated into single cell suspensions. The cells were then cultured in methylcellulose media for 4 days and BL-CFC colonies counted (Fig. 6) [42, 43]. Compared to RW4 cells we observed that scrambled and miR-24 KD clones had a reduced ability to make BL-CFCs. There was no difference in the production of BL-CFCs from scrambled and miR-24 shRNA expressing clones at d2.75 (Fig. 6A). From d3 EBs we observed a slight but significant increase (P<0.0007) in BL-CFCs obtained from miR-24 KD clones compared to scrambled control clones (Fig. 6B). However the number of BL-CFCs obtained from the miR-24 KD cells was lower than the numbers observed with wildtype cells.

To determine if the BL-CFC colonies were identical between controls and miR-24 KD cultures, we examined gene expression in the BL-CFC colonies. Twenty colonies from each culture were picked for generation of cDNA, and then individual gene expression was analyzed by Q-RT-PCR. Consistent with the EB results (Figs. 1C, 2B, 2C), there is a dramatic decrease in the expression of Gata1, Runx1, and Scl in the miR-24 KD BL-CFCs compared to control scrambled shRNA expressing clones (Fig. 6D). However endothelial genes Cdh5 (Ve-Cadherin), and Pecam1 were expressed similarly between control and miR-24 KD cultures. This suggests that there is a defect in the development of hemogenic endothelium, or the differentiation of hemogenic endothelium into blood. We attempted to determine if hemogenic endothelium was being produced by culturing Flk1+ cells, and analyzing by flow cytometry for the markers Tie2, and cKit according to the methods of Lancrin et al. [5]but were unable to generate sufficient number of differentiated cells to make any conclusions.

We also analyzed the expression of the transcription factor Etv2 to evaluate early hematopoietic progenitor generation. RNA was isolated from d3, d4, and d5 EBs. As previously observed Etv2 expression initially increases with wildtype EB differentiation and then decreases [21]. We observed a significant decrease in Etv2 expression between control clones and the miR-24KD clones at all time points (Fig. 6C). However Etv2 was reduced approximately 50% which was not as dramatic a reduction as observed with the downstream factor Scl (Fig. 2B).

**Fig 7:**
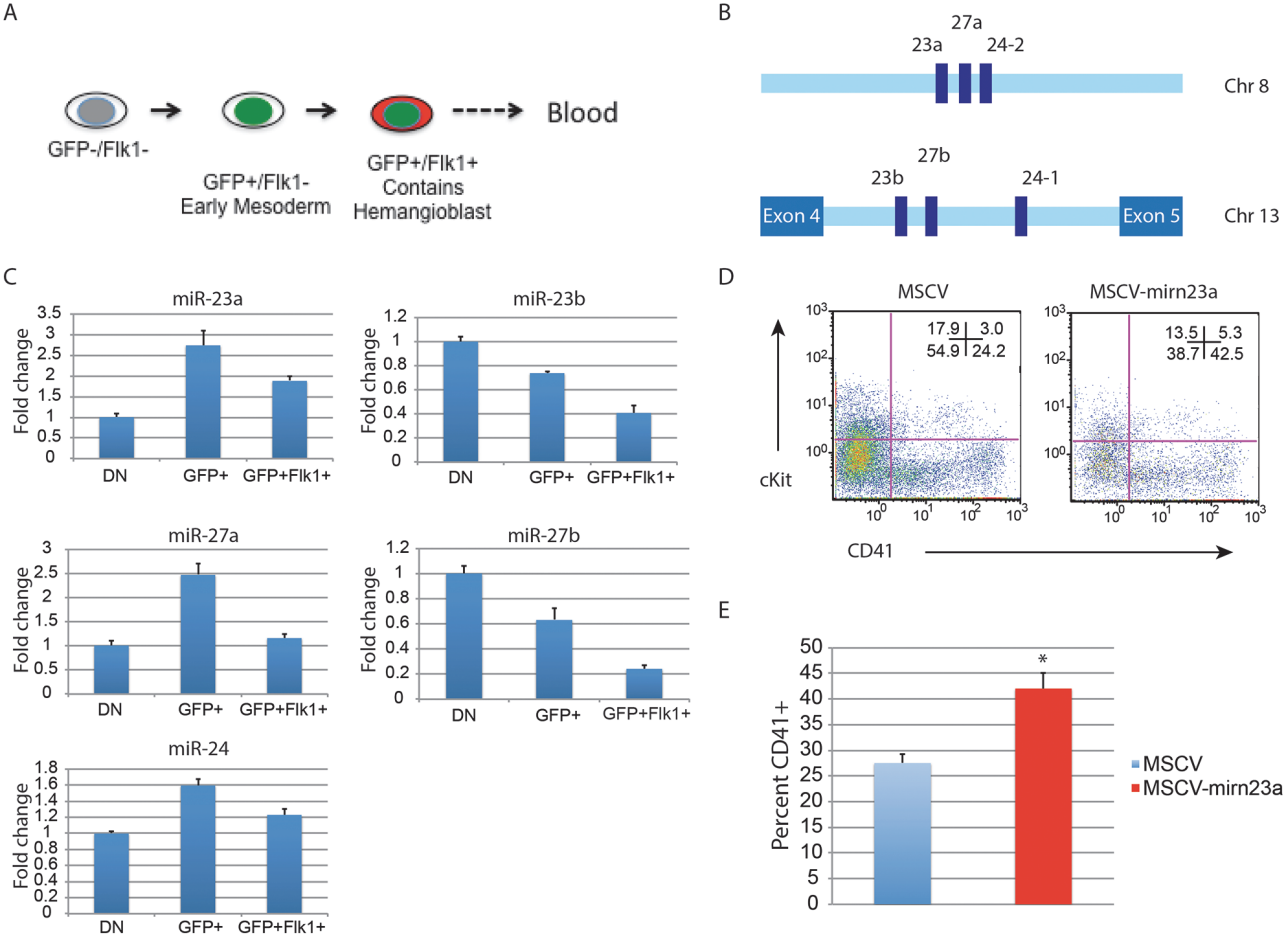
Expression of the gene encoding miR-24–2 enhances hematopoietic development in EBs. **A)** ESCs with GFP knocked in to the T locus (Brachyury) were differentiated for 4d into EBs. The EBs were dissociated into single cells suspensions, and sorted into GFP-Flk1-, GFP+/Flk1-, and GFP+Flk1+ fractions. **B)** The mirn23a miRNAs, miR-23a, 24–2, and 27a are clustered on mouse chromosome 8 and transcribed from an independent transcription unit. The mirn23b miRNAs, miR-23b, 24–1, and 27b are clustered on mouse chromosome 13 and are embedded in an intron of aminopeptidase O. **C)** RNA was isolated from the fractions diagrammed in panel A and used to evaluate the relative expression of the mature mirn23a, and mirn23b miRNAs in the fractions. Expression levels shown are relative to the GFP-/Flk1- (DN) fraction. **D)** D3 EB cells were infected with MSCV control or MSCV-mirn23a retrovirus, and reformed into EBs by hanging drop. The EBs were cultured an additional 5 days and contribution of the infected (GFP+) cells to the HPC population (CD41+, and CD41+cKit+) was evaluated. **E)** Three independent transductions, and subsequent differentiation were performed. The average percent of CD41+ HPCs is shown. The increase in CD41+ cells in the MSCV-mirn23a transduced cultures was significant with P<0.008 as determined by unpaired t-test.

**Expression of the gene encoding pre-miR-24–2 enhances hematopoietic development of d3 EB derived cells**. The BL-CFC (hemangioblast) is present in the T positive FLK1+ fraction of the differentiating EBs [11]. From d4 EBs generated from an ESC line with GFP knocked-in to the T locus we isolated T- (GFP^-^)/Flk1^-^, T^+^ (GFP^+^)/Flk1^-^, and T^+^ (GFP^+^)/Flk1^+^ cells by FACs (Figs. 7A, S5). RNA was prepared from the isolated cell population and the validity of the sort was examined by assaying the expression of genes known to be associated with each fraction (S5 Fig.)[11]. Q-RT-PCR was performed with RNA extracted from the isolated fractions to examine the relative levels of mature mirn23a/ mirn23b miRNAs: miR-24, mir-23a, miR23b, miR-27a, and miR-27b (Fig. 7B, 7C). MiR-24 was enriched in the mesoderm T^+^ (GFP^+^)/Flk1- fraction. MiR-24 decreased in the T^+^ (GFP^+^)/Flk1^+^ cells, the least expression was observed in the double negative fraction. A similar pattern was seen with miRs-23a, and 27a. However expression of miR-23b and miR-27b was highest in the double negative fraction with lowest levels expressed in the T^+^ (GFP^+^)/Flk1^+^ fraction.

Since endogenous mirn23a gene expression increased in mesoderm tissue fated to become hematopoietic tissue, we overexpressed it via retrovirus in differentiating ESCs to determine if exogenous expression could enhance hematopoietic development. We hypothesized that in order for mirn23a to enhance hematopoietic development that it needs to be expressed in the newly formed mesoderm. To express mirn23a after early mesoderm was formed, we first differentiated wildtype ESCs into EBs for 3d without viral infection. EBs were then disaggregated into single cell suspension and infected with control (MSCV) retrovirus or the mirn23a-expressing virus. EBs were reformed by hanging drop and allowed to differentiate for additional 5 days. Contribution of the infected cells (GFP+) to the CD41+ HPC population was then assayed by flow cytometry. Expressing the mirn23a cluster at a later stage in development significantly enhanced the development of CD41+ HPCs (Fig. 7D). Three independent transductions/ differentiations were performed demonstrating that mirn23a expression increased HPCs production (P<0.008) (Fig. 7E).

### Expression of MiR-24 target Trib3 inhibits hematopoietic development of ESCs

**Fig 8:**
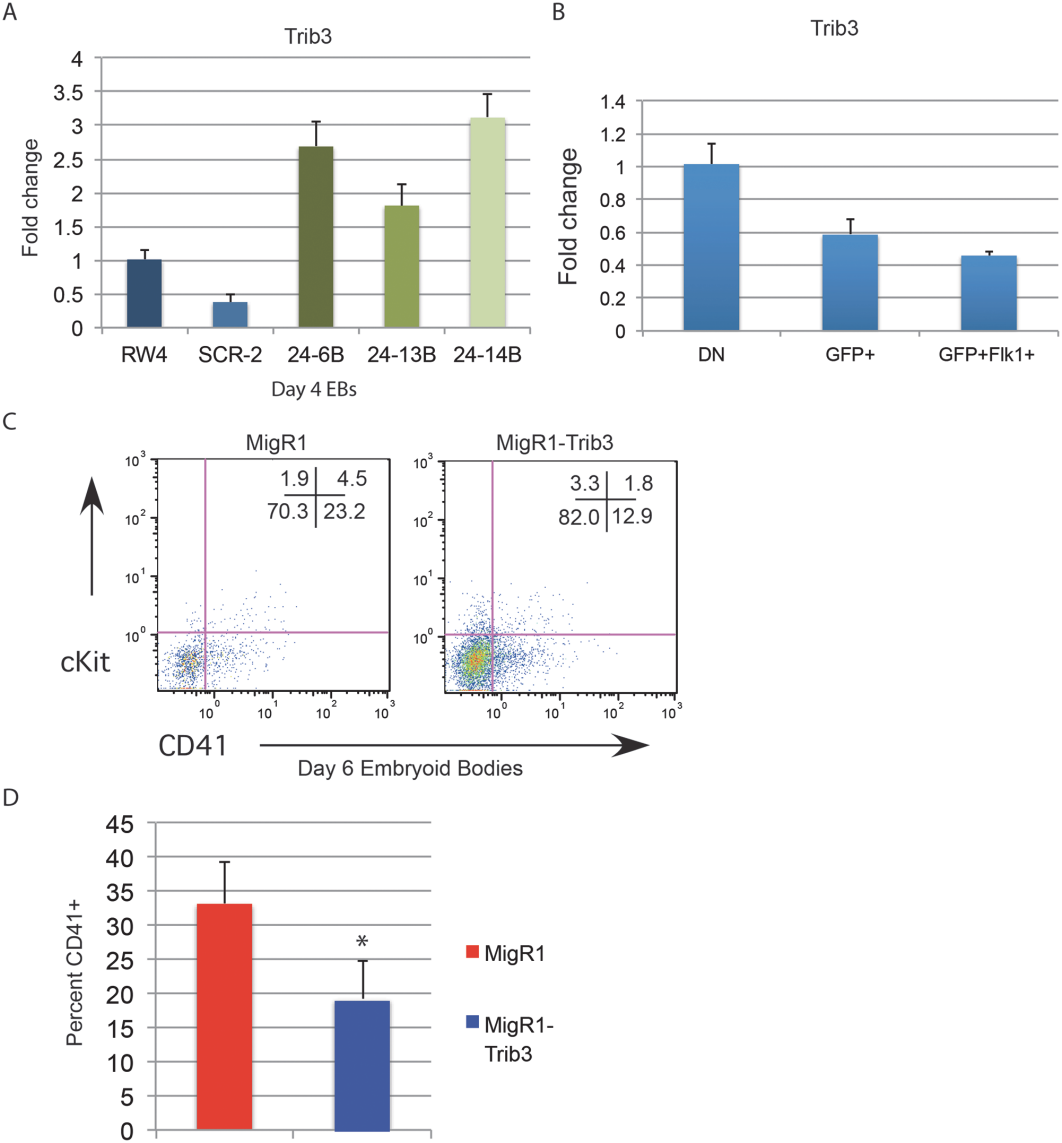
Expression of miR-24 target Trib3 impairs hematopoietic development of ESCs. **A)** Trib3 gene expression in d4 EBs derived from the indicated ESC clones. The increase in expression observed in the miArrest-24 clones compared to uninfected and miArrest-SCR infected RW4 cells was significant with a P value of <0.00002 as determined by unpaired t-test. **B)** Relative Trib3 mRNA expression as determined by Q-RT-PCR of RNA isolated from d4 EBs fractionated as describe in Fig. 7. **C)** ESCs were taken out of LIF and spin-infected with either MigR1 or MigR1-Trib3 virus for 1.5h. Cells were then cultured as hanging drops for 48h to form EBs. EBs were then transferred to liquid culture and grown and additional 6d before flow cytometry analysis. Surface expression of the HPC markers CD41 and cKit were examined on GFP+ cells. **D)** Three independent infections were performed and the average percent of CD41+ HPCs observed is shown. The decrease in CD41+ cells induced by Trib3 expression is significant with a P value of <0.001.

Trib3 is a validated target of miR-24 in vascular smooth muscle cells [31]. Misregulation of Trib3 has the potential to influence hematopoietic differentiation through the BMP4/ Smad Pathway [32–34, 36]. To determine if miR-24 targeting of Trib3 plays a role in the early hematopoiesis, we examined expression of Trib3 in d4 miR-24 KD EBs. Consistent with Trib3 being a miR-24 target we observed a 2 to 3 fold increase in Trib3 mRNA in miR-24 KD EBs compared to controls (Fig. 8A, P<0.00002). Additionally, Trib3 has the opposite expression pattern in d4 fractionated EBs compared to miR-24(Fig. 8B). Trib3 is expressed highest in the non-mesoderm fraction, whereas miR-24 is more highly expressed in the T+ mesoderm fractions. To test whether this overexpression of Trib3 contributes to the block in hematopoietic development observed in miR-24 KD EBs, we expressed Trib3 in wildtype ESCs at the onset of differentiation, and assayed HPC development 6d later. Trib3 was expressed in ESCs via retroviral transduction of cells freshly withdrawn from LIF. Infected cells were identified by co-expression of GFP. HPC generation as evaluated by CD41 expression was reduced almost 2-fold by expression of Trib3 compared to ESCs infected with control virus (P<0.001, Fig. 8C, 8D). A similar inhibition of EB hematopoiesis was observed if we infected d3 EBs with Trib3 retrovirus, and then examined the CD41+ population after 5 additional days of EB culture (S6 Fig.). Additionally if we knocked down Trib3 expression in ESCs with lentiviral delivered shRNA, we observed the opposite effect on hematopoiesis. EBs derived from Trib3 shRNA-expressing ESCs had an increased production of CD41+ cells, compared to EBs derived from ESCs expressing a non-targeting shRNA (S7 Fig.).

## Discussion

Using two different lentiviral vectors delivering distinct shRNAs targeting miR-24, we observed that miR-24 is required for hematopoietic development from ESCs. The absence of hemoglobinized cells and greatly reduced expression of hematopoietic transcription factors Sfpi1 and Gata1 indicate that blood development is greatly impaired in EBs generated from miR-24 KD ESCs. Flow cytometry revealed that miR-24 is required early in development as we observed a reduction in the number of CD41+ HPCs developing within EBs when miR-24 is antagonized. Consistent with the flow cytometry, we detect reduced expression of transcription factors essential for early hematopoiesis: Scl, and Runx1.

The defect in hematopoiesis is not due to a general inability of the miR-24 ESCs to differentiate. ESCs with antagonized mR-24 express endoderm, ectoderm, and mesoderm genes normally compared to control ESCs. Cell surface expression of FLK1, and PDGFRα indicates that lateral plate, and paraxial mesoderm is made in knockdown cells.

However decreased expression of Twist1 in miR-24 KD EBs suggests that there is a reduction in lateral plate mesoderm. This may be due to the decreased production of the lateral plate derived blood cells. Lateral plate mesoderm also gives rise to vasculature and cardiac tissue. We observed no deficits in the generation of cardiac tissue. Vascular development may be slightly impaired as we observe an early decrease in expression of the endothelial gene Pecam1 and a slight decrease in class 4 sprouts in an EB vascular sprout assay. Due to the decreased expression of Scl, it was surprising that we did not observe enhanced cardiac muscle development. Scl has been demonstrated to be a repressor of early cardiac muscle development [44, 45]. However, only overexpression of Scl has been reported to affect ESC cardiac development. Loss of Scl resulted in increased cardiac differentiation in vivo in the mouse, but this may differ from reduced expression we see in our system. In addition, our assay may not be sensitive enough to detect changes in cardiac development. However, since we see a dramatic impairment in HPC production, but little or no impairment of other mesoderm derived tissue the data demonstrates that miR-24 is not needed generally for differentiation.

The phenotype we observed from miR-24 KD ESCs cells suggest an early requirement for miR-24 in embryonic hematopoiesis, potentially in the development or function of the hemangioblast. Results from the BL-CFC assays suggest that miR-24 is not required for the commitment of mesoderm to the hemangioblast, as we did not observe a requirement for miR-24 in BL-CFC generation. However, the decreased expression of Etv2, and Runx1, along with the dramatic reduction in Scl suggests that there is a defect in differentiation downstream of the hemangioblast. Expression of endothelial genes Pecam1, and Cdh5 was unaffected in miR-24 KD BL-CFC suggesting a potential defect in the development of hemogenic endothelium from the BL-CFC, or a defect in the ability of hemogenic endothelium to produce HPCs.

MiR-24 was previously shown to target Trib3 in vascular smooth muscle cells [31]. Consistent with it being a miR-24 target in EBs, Trib3 levels increase in d4 miR-24 KD EBs. Additionally Trib3 expression goes down with mesoderm commitment in wildtype EBs, as miR-24 levels go up. To determine if increased Trib3 contributes to the phenotype we retrovirally transduced differentiating wildtype ESCs with Trib3 retrovirus to mimic the overexpression seen in miR-24 KD ESCs cells. Trib3 expression significantly inhibited production of HPCs from infected ESCs compared to ESCs infected with control virus. The inhibition of hematopoiesis is not as dramatic as what is observed with the antagonism of miR-24, however it suggests that Trib3 downregulation contributes to miR-24’s ability to promote early hematopoiesis. Consistent with Trib3 being a negative regulator of hematopoiesis, we observed an increase in CD41+ HPC production when Trib3 was antagonized in ESCs.

Trib3 is a member of the Tribbles family of proteins, which includes Trib1, and Trib2. Trib3 is a negative regulator of the serine threonine kinase Akt [46]. In the zebrafish embryo, PI3K/ Akt signaling is necessary for hematopoiesis and angiogenesis [47]. Additionally, Trib3 negatively regulates Smurf1, the E3 ubiquitin ligase, which targets Smads 1 and 5 proteins for destruction [31, 32]. BMP signaling through Smads is critical component of the gene regulatory network that drives early hematopoietic differentiation. Smad1 is required specifically at different stages of early development of mesoderm into hematopoietic progenitors with increased expression of Smad1 in nascent ESC derived mesoderm increasing the development of hemangioblast, whereas increased expression in the hemangioblast and/or hemogenic endothelium blocks hematopoietic differentiation [33, 36]. The distinct temporal effects of Smad1 on hematopoiesis are similar to what we observe with miR-24, which will inhibit hematopoiesis when expressed at the beginning of ESC differentiation, but increases hematopoiesis when expressed at d3 of differentiation. Increased Trib3 in miR-24 KD ESCs may disrupt BMP signaling inhibiting development of HPCs. It will be important to determine if disruption of PI3 kinase and/or Smad signaling contributes to the block in hematopoietic development we observe. Identification of other targets of miR-24 that may be upregulated in EBs to block hematopoiesis is also critical.

Previous to this study no miRNA had been observed to regulate hematopoiesis at this early point in mammalian ontogeny. However, a requirement for miRNAs in embryonic hematopoiesis was recently shown in Xenopus [30]. Morpholino knockdown of the microprocessor complex member Dgcr1 results in a global knockdown of mature miRNAs in the Xenopus embryo, and ablates production of the hemangioblast and hemogenic endothelium as evaluated by decreased expression of Runx1 and Flk1. Candidate miRNAs in regulating embryonic hematopoiesis were identified by examining miRNAs enriched in AGM HPCs isolated from the d11.5 mouse embryos. As a secondary screen, AGM HPC miRNAs were subjected to bioinformatics analysis to determine if any of these miRNA genes had ChIP-seq peaks for hematopoietic transcription factors binding within 5 kb of the pre-miRNA locus. The top candidate in this screen was miR-142. Interestingly in their analysis miR-24 was enriched in the AGM HPCs, and was the third top ranked miRNA gene in the ChIP-seq analysis. Expression of a mature miR-142, but not miR-24 was able to rescue hemangioblast development in Dgcr1 knockdown embryos, however miR-142 was unable to rescue hemogenic endothelium development. These results appear consistent with what we have observed that miR-24 is not required for hemangioblast development, but is needed for subsequent hematopoietic differentiation.

Understanding the gene regulation network of stem cells is critical for the development of protocols for directing the differentiation of stem cells into tissues needed for transplant. Unfortunately, it is still difficult to match donors with transplant recipients, with many patients unable to obtain life-saving tissue that is needed. The generation of induced Pluripotent Stem Cells (iPSCs) from adult cells holds the promise of generating abundant patient specific tissues for transplant [48–50]. Efficient and robust iPSC differentiation, or possibly other patient-derived adult stem cells could lead to production of hematopoietic stem cells (HSCs) for transplantation therapy to treat hematological disorders. MiRNAs are attractive drug targets due to their small size, which may make them amenable to making small molecule mimics as well as making antagonists [51]. MiR-24 is the first miRNA to be involved in this early step of differentiation of mammalian mesoderm to tissue that will give rise to HPCs. Manipulating miR-24 or its downstream targets potentially could be used for directing the differentiation of nascent mesoderm produced from pluripotent stem cells.

## Materials and Methods

### Lentivirus and retrovirus production

ShRNA antagonist of miR-24 (miArrest miRNA inhibitor) or scrambled non-targeting shRNA was expressed in a lentiviral vector co-expressing mCherry and puromycin resistance (GeneCopoeia, Rockville, MD). For supplementary experiments miR-ZIP vectors to deliver an independent miR-24 targeting shRNA and scrambled shRNA were used (System Biosciences, Mountain View, CA). pLKO.1 lentiviral plasmids expressing Trb3 ShRNA antagonists were obtained from Thermo Scientific (Waltham, MA). pVSV-G, pMDL, and pRSV-REV plasmids (National Gene Vector Biorepository, Indianapolis, IN) were co-transfected with lentiviral plasmid into 293FT cells with Lipofectamine 2000 Transfection Reagent (Invitrogen, San Diego, CA). For retroviral production MigR1-EGFP, MigR1-Trib3, MSCV-EGFP, and MSCV-mirn23a retroviral plasmids were co-transfected into 293FT cells together with the retroviral packaging vector pCL-Eco (Imgenex) using Lipofectamine 2000 (Invitrogen). For both lentivirus and retrovirus production 48h and 72h post-transfection viral supernatants were harvested and concentrated with Centricon Plus-70 filters (Millipore). MigR1-Trib3 plasmid was kindly provided by Dr. Keyong Du, Tufts University, Boston, MA.

### Embryonic stem cells

RW4 ESCs (ATCC, Manassas, VA) were adapted from growth on mitomycin C-treated MEFs to growth on plates coated with 0.1% porcine skin gelatin (Sigma, St. Louis, MO). After passaging 3 times on gelatinized plates, cells were frozen or immediately used for viral infections. RW4 ESCs were maintained on gelatin coated plates in DMEM (Invitrogen, Carlsbad, CA), 15% fetal bovine serum (FBS, Hyclone, Pittsburgh, PA), 50 U/ml penicillin, and 50 ug/ml streptomycin, 1 X Non Essential Amino Acids, 2mM Glutamax, 55 μM 2-mercaptoethanol (BME), and 1000U/ml LIF (ESGRO, Millipore, Billerica, MA). Dulbecco’s Modified Eagle Medium (DMEM) and media additives unless otherwise indicated were obtained from Invitrogen (Carlsbad, CA). Gelatin adapted GFP-Brachyury ESCs were obtained from Valerie Kouskoff (Cancer Research UK Manchester Institute), and were cultured as the RW4 cells.

### Differentiation of ESCs

Embryoid bodies were generated from ESCs by either liquid culture or methylcellulose culture. For liquid culture ESCs were washed out of LIF containing media, and plated onto 10 cm petri plates (Fisher Scientific, Pittsburgh, PA) at 10,000 cells/ml in ESC media without LIF (Differentiation media). ESCs were harvested at the indicated times. For methylcellulose differentiation, 10,000 ESCs were washed out of LIF and subsequently cultured in 1.5 ml 0.9% methylcellulose-base medium (Stem Cell Technologies, Vancouver, CA) supplemented with 10% FBS (Hyclone), 5% protein-free hybridoma medium-II (Invitrogen, Carlsbad, CA), mIL-1 (5 ng/ml), mIL-3 (5 ng/ml), SCF (10 ng/ml), mGM-CSF (5 ng/ml) and hEPO (3 U/ml) at 37°C and 5% CO_2_ for 14 days. All cytokines were obtained from Invitrogen except for hEPO, which was obtained from Stem Cell Technologies.

### Vascular sprout assays

2000 ESCs/ 35mm dish were differentiated into EBs for 11 days in 1.0% Base Methylcellulose (M3120, Stem Cell Technologies, Vancouver, CA), 15% FBS (Hyclone), 10ug/ml insulin (Sigma, St. Louis, MO), 100ug/ml mFGF2 (Invitrogen, Carlsbad, CA), 50ug/ml VEGF (Biolegend, San Diego, CA), 10 ng/mL mIL-6 (Invitrogen, Carlsbad, CA), 2 U/mL hEPO (Stem Cell Technologies, Vancouver, CA), 450 μM monothioglycerol (MTG, Sigma, St. Louis, MO). 125 d11 EBs were plated onto 35mm plates in ES-Cult Endothelial Collagen Medium (Stem Cell Technologies, Vancouver, CA) containing 50 ng/ml mVEGF, 100 ng/ml mFGF2, 10 ng/mL mIL-6, and 2 U/mL hEPO. Sprout formation was assessed 4 days later. EBs were scored in blinded fashion according to 4 standard classes based on vascular sprout formation: I- no sprout formation, II- few sprouts, III- many sprouts but no network, IV- many sprouts with network [41].

### Cardiac differentiation assays

EBs were initially generated by hanging drops. ESCs were plated 25,000-cells/ ml in ESC media minus LIF in 20ul drops hanging from an inverted 15cm plate lid. The 15cm plate contained 10ml H20 to keep the chamber humidified. EBs were cultured for 5d, and then individual EBs were transferred to an individual well of a 96 well tissue culture plate. After 5d of culture, each well was examined for the presence of beating cells indicating the presence of cardiac tissue.

### BL-CFC assay

Primary EBs were generated from ESCs by liquid culture onto 10 cm non-tissue culture petri plates (Fisher Scientific, Pittsburgh, PA) at 5,000 cells/ml in Iscove’s Modified Dulbecco Medium (Life Technologies, Grand Island, NY) supplemented with 15% differentiation fetal bovine serum (Stem Cell Technologies, Vancouver, BC, Canada), 5% protein-free hybridoma medium-II (Life Technologies), 200 ug/mL iron-saturated holo-transferrin (Sigma, St Louis, MO), 50 ug/mL ascorbic acid (Sigma), 450 uM monothioglycerol (Sigma), 2mM glutamine(Life Technologies) and 100ug/mL penicillin/streptomycin (Life Technologies). Primary EBs was differentiated at 37°C and 5% CO2 for 2–4 days.

At d2.75 or d3, the primary EBs from liquid culture were harvested and digested with Accumax (Millipore, Billerica, MA) and further dissociated into single cell suspension by passaging with a p200 pipet tip several times. For hemangioblast assays, 12,500 cells/mL in 35mm dishes were differentiated into BL-CFC colonies for 3–4 days in 0.9% Base Methylcellulose (M3120, Stem Cell Technologies, Vancouver, CA) supplemented with 10% differentiation fetal bovine serum (Stem Cell Technologies, Vancouver, BC, Canada), 200 ug/mL iron-saturated holo-transferrin (Sigma, St Louis, MO), 50 ug/mL ascorbic acid (Sigma), 450 uM monothioglycerol (Sigma), 2mM Glutamax (Life Technologies, Grand Island, NY), 100ug/mL penicillin/streptomycin (Life Technologies), 20% D4T endothelial cell conditioned media (Gift from Diana Ramirez, Case-Western University, Cleveland, OH), 100 ng/mL SCF (Life Technologies) and 5ng/mL VEGF (Biolegend, San Diego, CA). BL-CFC colonies were assessed 3–4 days later.

For gene expression analysis 20 BL-CFCs per genotype were picked at d4 of differentiation into PBS and pelleted by centrifugation. Cells were lysed, and used for preparation of cDNA with a Cells-to-Ct kit (Ambion) according to manufacturers instructions. The cDNA was then subjected to quantitative PCR using gene specific primers and fluorescently labeled probe as described below.

### Hematopoietic colony assay

ESCs were differentiated into EBs by methylcellulose culture as described above. After 9 days, EBs were harvested and disaggregated with trypsin and mechanical shearing with a 21-gauge needle. Cells were then replated into Methocult GF 3434 methylcellulose media (Stem Cell Technologies). The number of cells plated was equal to the number of cells in 50 EBs derived from wild-type ES cells. Number and colony type was enumerated 7 days later.

### Viral infection of ESCs

For miR-24 knockdown studies, RW4 ESCs were plated at 100,000 cells per well of a 6 well plate 24h pre-infection. The next day media was replaced with 1.5ml ESC media containing lentivirus. Cells were spin infected for 1.5h. Media was replaced the next day. 48h post-infection cells were split onto 10cm plates in ESC media containing 5ug/ml puromycin (Invitrogen, Carlsbad, CA). Ten to 12 days later resistant, colonies were isolated. Clones were tested for knockdown of miRNA expression using miR-Taqman assays as described below.

For retroviral infection of ESCs, cells were trypsinized and washed out of LIF containing media with PBS (2.7mM KCl, 1.8mM KH_2_PO_4_, 137mM NaCl, 10mM Na_2_HPO_4_, pH 7.4). 50,000 cells were replated in 1.5 ml of differentiation media containing retroviral supernatant and centrifuged at 2125 x g for 90 minutes. 1.5 ml of differentiation media was added to the cell. The cell suspensions were then used to form EBs by the hanging drop method. All 3mls of cell suspension were plated as 20ul drops on an inverted 15cm tissue culture plate lid. The lid was then replaced onto a petri plate and incubated in the tissue culture incubator for 2 days to allow for formation of EBs. After 2d the EBs were transferred to a 10 cm petri plate containing 7 ml of differentiation media. EBs were cultured for an additional 4 days, and differentiation assayed by flow cytometry.

For infection of EBs cells, single cell suspensions were prepared from d3 EBs prepared in liquid culture as described above. EBs were washed in PBS and disaggregated into single cell suspensions by incubating with 1ml of Accumax (Millipore, Billerica, MA) at 37°C for 30 minutes. To further break up the EBs, the cell solution was pipetted up and down in a 2ml pipet with a p200 tip at the end. Cells were spun down and resuspended in 1.5 ml of differentiation media. EB cells were then virally infected and reformed into EBs by hanging drop as described above. EBs were examined for hematopoietic differentiation after 5 days.

### Quantitative Reverse-Transcriptase Polymerase Chain Reaction (Q-RT-PCR)

Total RNA was prepared using TRIzol (Life Technologies, Grand Island, NY) according to the manufacturer’s protocol. For mRNA gene expression complementary DNA (cDNA) was prepared by reverse transcribing 1ug of total RNA using the High Capacity cDNA Reverse Transcriptase Kit according to manufacturer’s protocol (Life Technologies). Gene specific primer sets with fluorescent probes were obtained from IDT (Coralville, IA). ΔΔC_T_ calculations were used to normalize signal versus GAPDH as the control. For miRNA analysis, we used miR-specific reverse transcription primers and Taqman primers obtained from Life Technologies. Expression level of miR-24 was normalized to sno202 snRNA expression. All experiments were performed in triplicate using BioRad CFX96 C1000 System (BioRad, Hercules, CA).

### Flow cytometry

EBs were pelleted by centrifugation and washed with PBS. EBs were disaggregated by the addition of 1ml Accumax cell dissociation buffer (Millipore, Billerica, MA) and incubated at 37°C for 30 minutes. Single-cell suspensions were collected and washed with PBS and incubated with the indicated antibodies. CD41-PE, FLK1-PE, ckit-APC, PDGFRα-biotin, and avidin-APC-Cy7 were obtained from Biolegend (San Diego, CA). Stained cells were subsequently assessed using Beckman Coulter FC500 Flow Cytometer (Brea, CA) and data was analyzed using Flowjo software (Tree Star, Ashland, OR).

## Supporting Information

S1 Fig MiArrest-24 shRNA infection decreases the expression of miR-24 miRNA. **A)** Expression of miR-24 in undifferentiated RW4 clones infected with MiArrest-Scr (scrambled shRNA) or miArrest-24 (shRNA antagonizing miR-24) **B)** Average miR-24 expression in miArrest-Scr, and miArrest-24 infected ESC clones at the indicated days of differentiation (Removal of LIF). **C)** Average expression of mirn23a cluster miRNAs in undifferentiated miArrest-Scr, and miArrest-24 infected ESC clones.
(TIF)

S2 Fig Hematopoietic colonies production form Embryoid Bodies. Single cell suspensions were prepared from 9d EBs. Cells corresponding to 50 EB equivalents were plated into methylcellulose containing hematopoietic cytokines (Methocult M3434, Stem Cell Technology). Hematopoietic colonies were counted and scored 7d later. Data is average colony numbers obtained from 4 independent scrambled shRNA clones, and 3 miR-24 shRNA clones.
(TIF)

S3 Fig Antagonizing miR-24 is ESCs with a distinct shRNA delivered by miRZIP vectors results in a hematopoietic defect. **A)** MiR-24 expression in undifferentiated miRZIP-miR-24 shRNA ESC clones (24–6, 24–7) compared to ESC clone (SCR-2) infected with miRZIP vector coding for a scrambled non-targeting shRNA. **B)** 14d methylcellulose differentiation of the indicated miRZIP clones into EBs. **C)** Flow cytometry analysis of CD41 and cKit cell surface expression on single cells isolated from 6d EBs generated from the indicated ESC clones.
(TIF)

S4 Fig Time course of development of CD41+ HPCs from RW2 and miArrest-24 infected ESCs. Flow cytometry analysis of CD41 and ckit cell surface expression on single cell suspensions prepared from EBs. Single cell suspensions were prepared from EBs derived from RW4, or a miArrest-24 infected ESC clones isolated at d3, d4, d5, and d6 post removal of LIF.
(TIF)

S5 Fig Fractionation of d4 EBs. ESCs with GFP knocked in to the T locus (Brachyury) were differentiated for 4d into EBs. The EBs were dissociated into single cells suspensions, and sorted into GFP-Flk1-, GFP+Flk1-, and GFP+Flk1+ fractions. The GFP versus Flk1 FACs plot shows the 3 gates of cells that were collected. To verify that the fractions were sorted properly Q-RT-PCR was performed on RNA isolated from the fractionated cells. Gene expression agreed with previously published data using this ESC line [11].
(TIF)

S6 Fig Trib3 impairs hematopoietic development when expressed at d3 of EB differentiation. A) Single cell suspensions were prepared from d3 EB cells and infected with MigR1 control or MigR1-Trib3 retrovirus. EBs were reformed by hanging drop, and cultured an additional 5 days. Contribution of the infected (GFP+) cells to the HPC population CD41+, and CD41+cKit+ was evaluated by flow cytometry. Results from 2 independent infections/ differentiations are shown.
(TIF)

S7 Fig Knockdown of Trib3 enhances hematopoietic differentiation. RW4 ESCs were infected with empty vector (pLKO.1), Trib3 shRNA (KD), or non-targeting shRNA expressing lentiviruses. Infected cell clones were generated by selection in puromycin. 1 pLKO.1, 2 independent Trib3 shRNA, and 1 non-targeting (NT) clones were examined. **A)** Quantitative RT-PCR assaying Trib3 expression in the isolated clones. B) Flow cytometry analysis of CD41 and cKit (CD117) cell surface expression on single cells isolated from EBs generated from the indicated ESC clones. CD41+cKit- population contains primitive HPCs and CD41+cKit+ population contains primitive and definitive HPCs.
(TIF)
