# Supplementary figures and images for: MiR-24 Is Required for Hematopoietic Differentiation of Mouse Embryonic Stem Cells
Source: PLoS Genet. 2015 Jan 29;11(1):e1004959. doi: 10.1371/journal.pgen.1004959 (PMC4310609; doi:10.1371/journal.pgen.1004959)

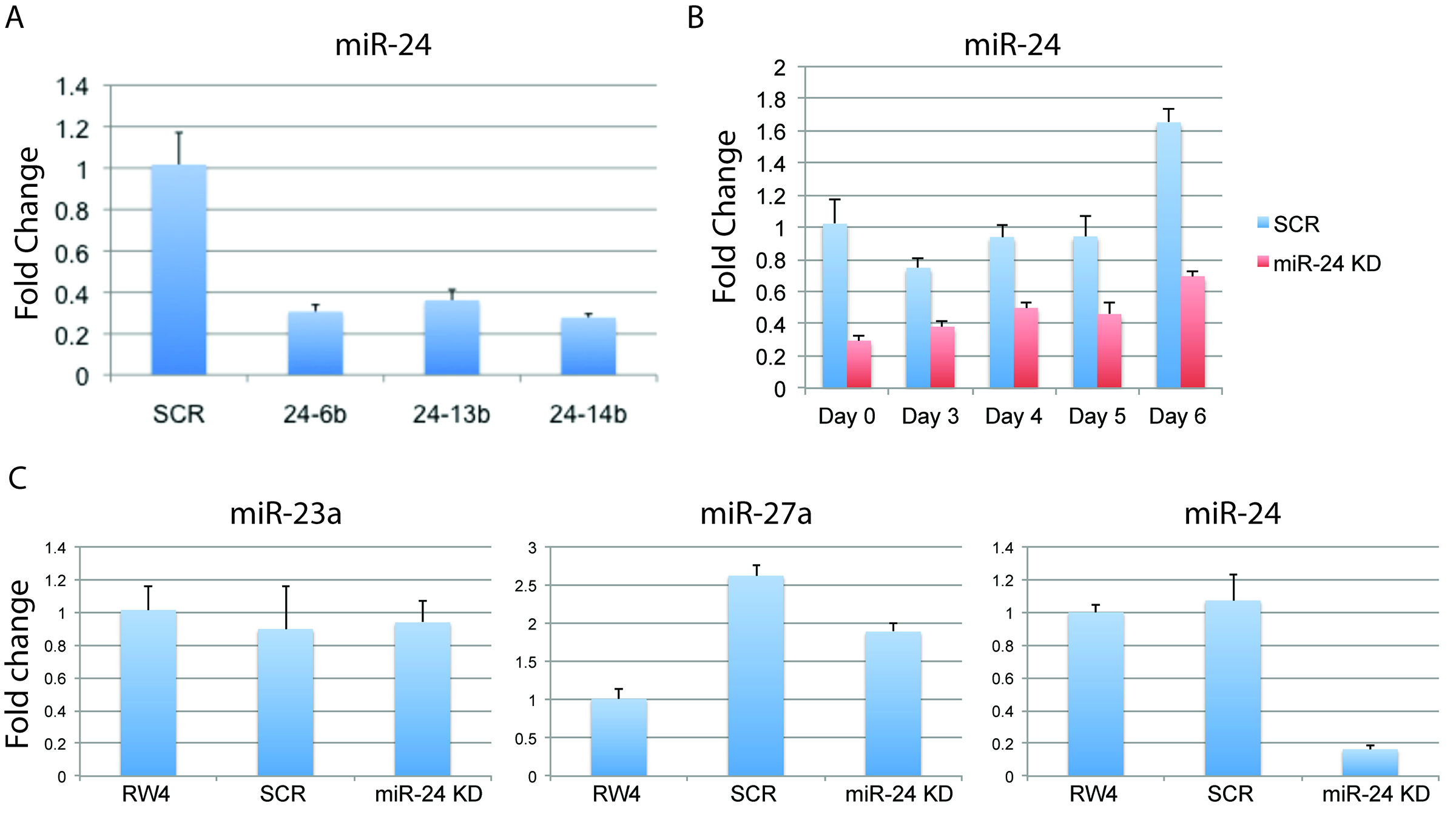

Supplement: S1 Fig — A) Expression of miR-24 in undifferentiated RW4 clones infected with MiArrest-Scr (scrambled shRNA) or miArrest-24 (shRNA antagonizing miR-24) B) Average miR-24 expression in miArrest-Scr, and miArrest-24 infected ESC clones at the indicated days of differentiation (Removal of LIF). C) Average expression of mirn23a cluster miRNAs in undifferentiated miArrest-Scr, and miArrest-24 infected ESC clones. (TIF) [file pgen.1004959.s001.tif]

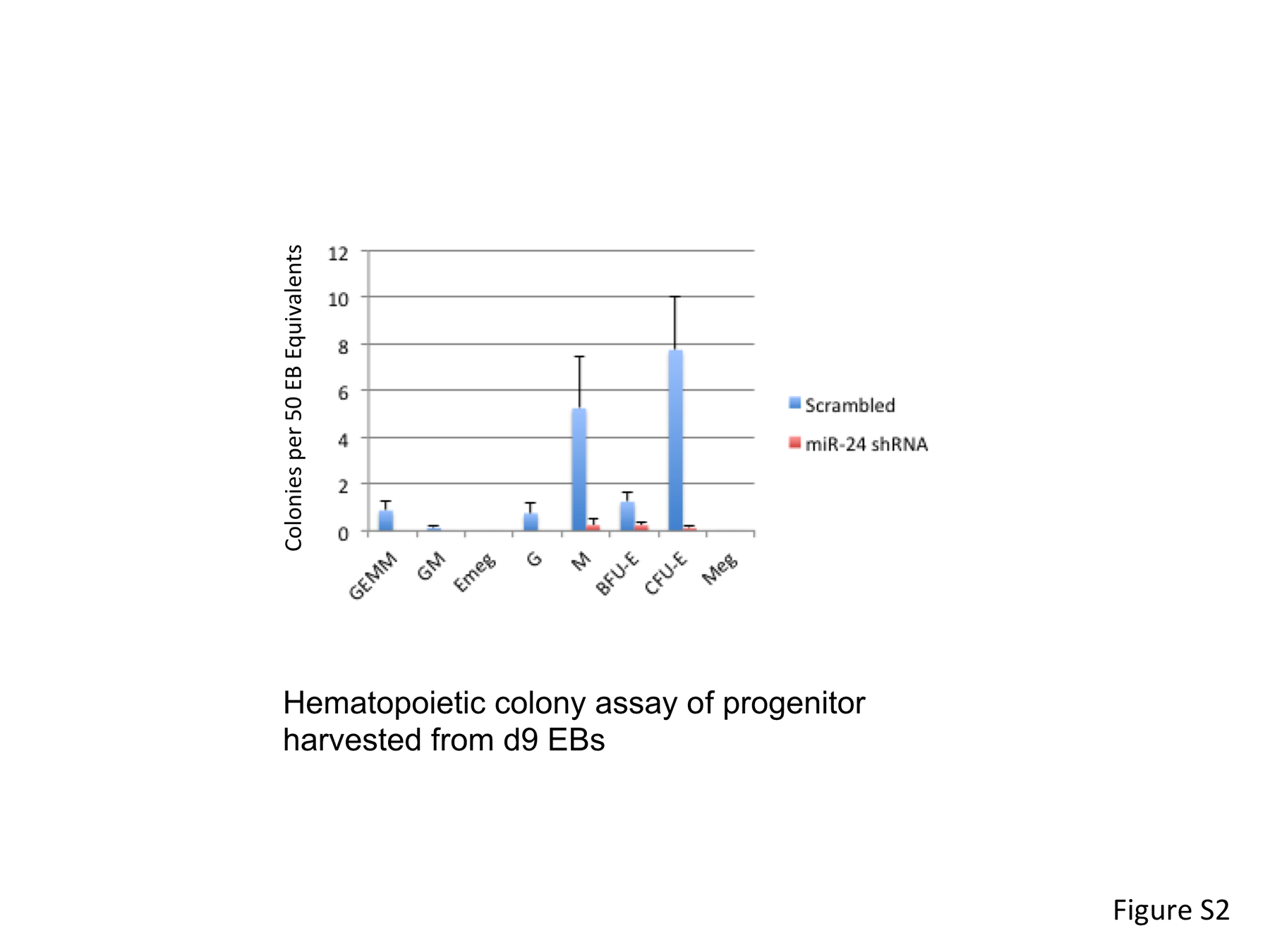

Supplement: S2 Fig — Single cell suspensions were prepared from 9d EBs. Cells corresponding to 50 EB equivalents were plated into methylcellulose containing hematopoietic cytokines (Methocult M3434, Stem Cell Technology). Hematopoietic colonies were counted and scored 7d later. Data is average colony numbers obtained from 4 independent scrambled shRNA clones, and 3 miR-24 shRNA clones. (TIF) [file pgen.1004959.s002.tif]

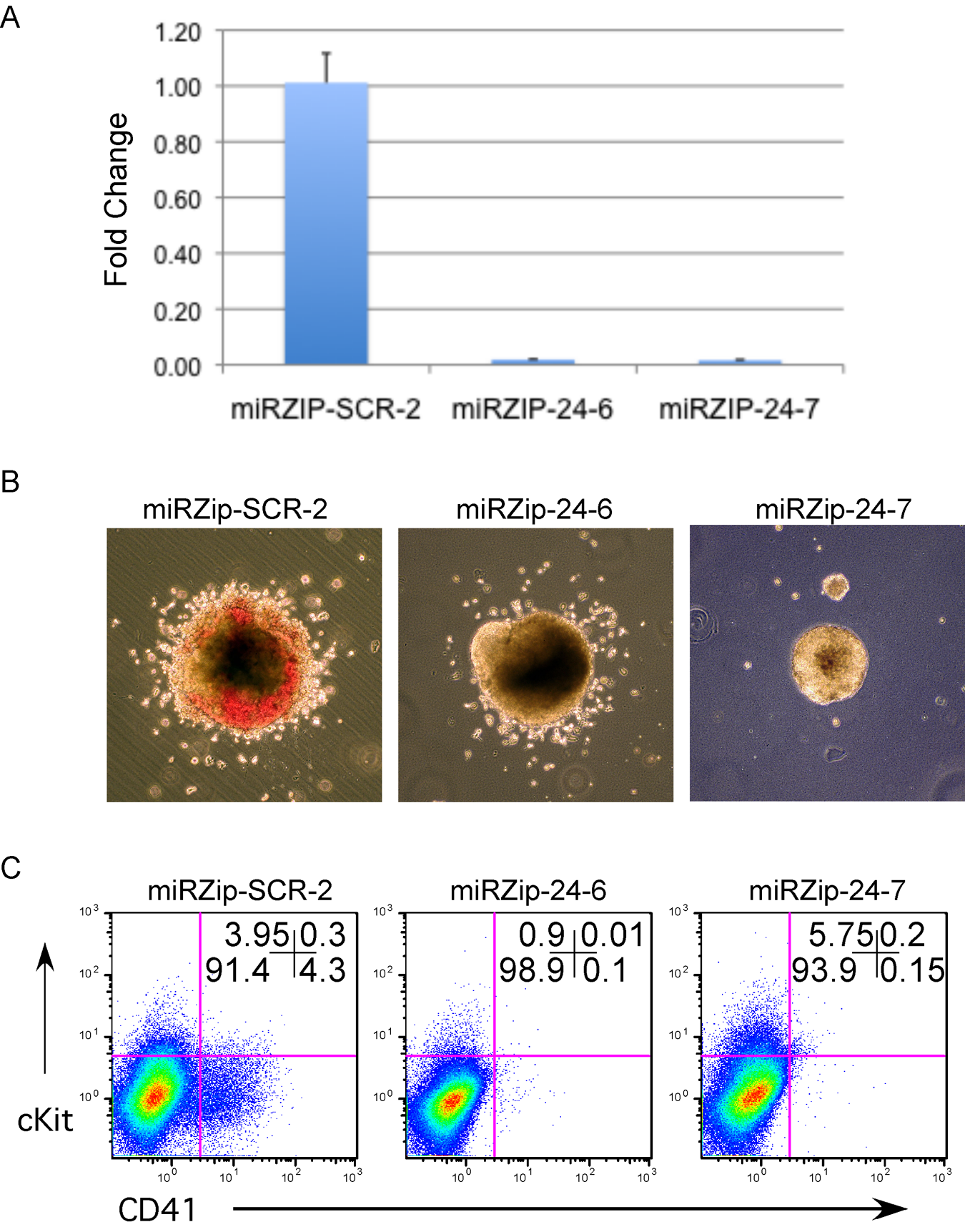

Supplement: S3 Fig — A) MiR-24 expression in undifferentiated miRZIP-miR-24 shRNA ESC clones (24–6, 24–7) compared to ESC clone (SCR-2) infected with miRZIP vector coding for a scrambled non-targeting shRNA. B) 14d methylcellulose differentiation of the indicated miRZIP clones into EBs. C) Flow cytometry analysis of CD41 and cKit cell surface expression on single cells isolated from 6d EBs generated from the indicated ESC clones. (TIF) [file pgen.1004959.s003.tif]

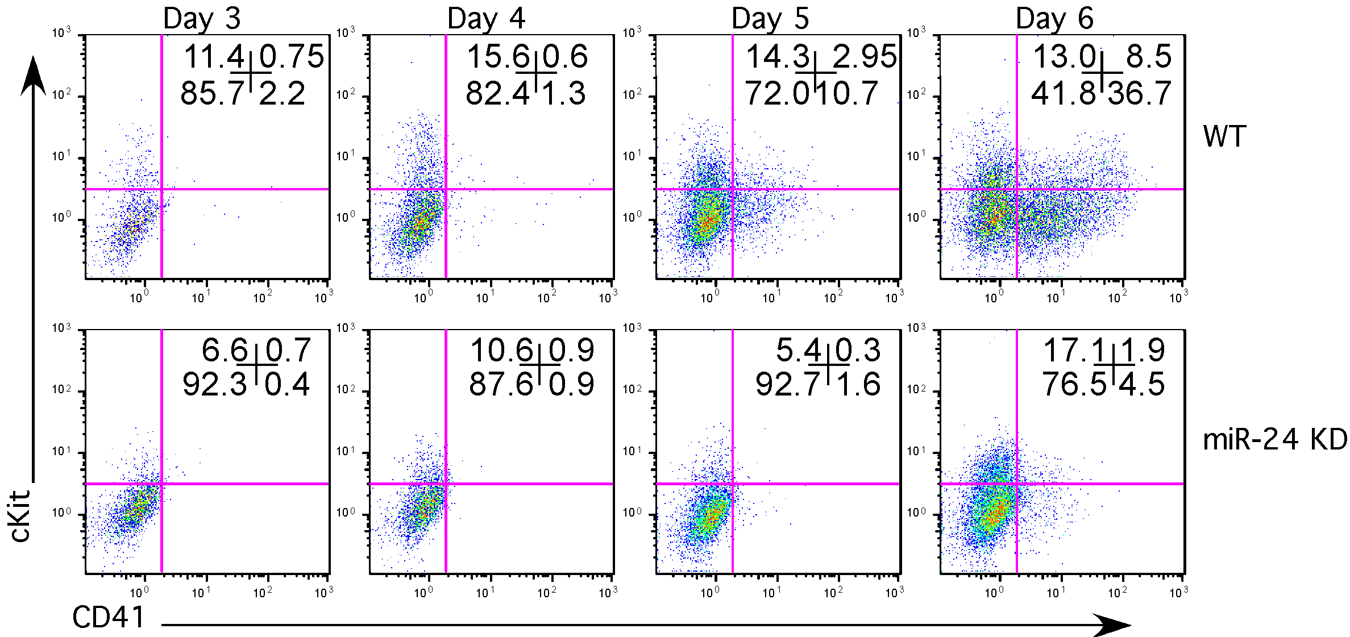

Supplement: S4 Fig — Flow cytometry analysis of CD41 and ckit cell surface expression on single cell suspensions prepared from EBs. Single cell suspensions were prepared from EBs derived from RW4, or a miArrest-24 infected ESC clones isolated at d3, d4, d5, and d6 post removal of LIF. (TIF) [file pgen.1004959.s004.tif]

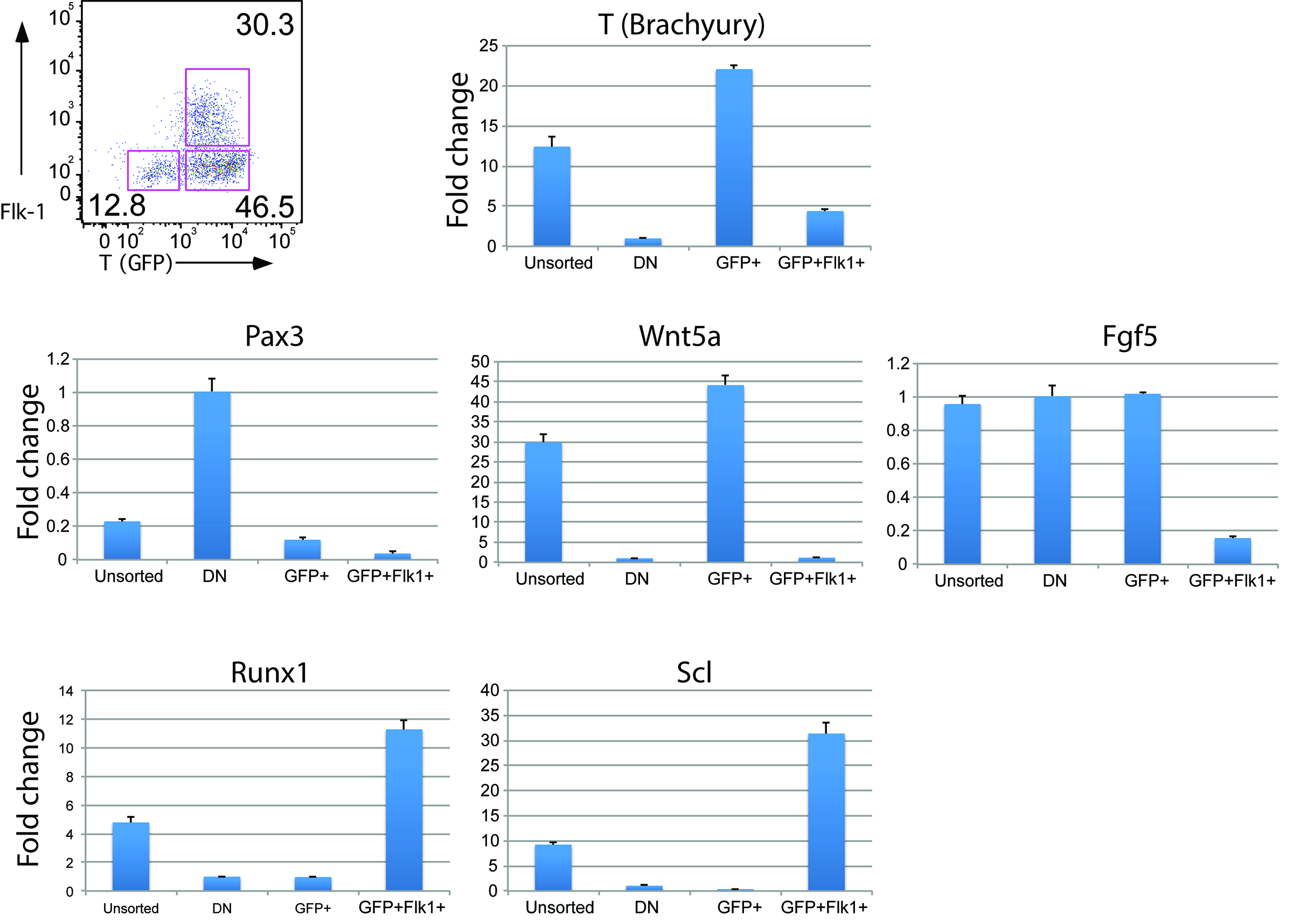

Supplement: S5 Fig — ESCs with GFP knocked in to the T locus (Brachyury) were differentiated for 4d into EBs. The EBs were dissociated into single cells suspensions, and sorted into GFP-Flk1-, GFP+Flk1-, and GFP+Flk1+ fractions. The GFP versus Flk1 FACs plot shows the 3 gates of cells that were collected. To verify that the fractions were sorted properly Q-RT-PCR was performed on RNA isolated from the fractionated cells. Gene expression agreed with previously published data using this ESC line [11]. (TIF) [file pgen.1004959.s005.tif]

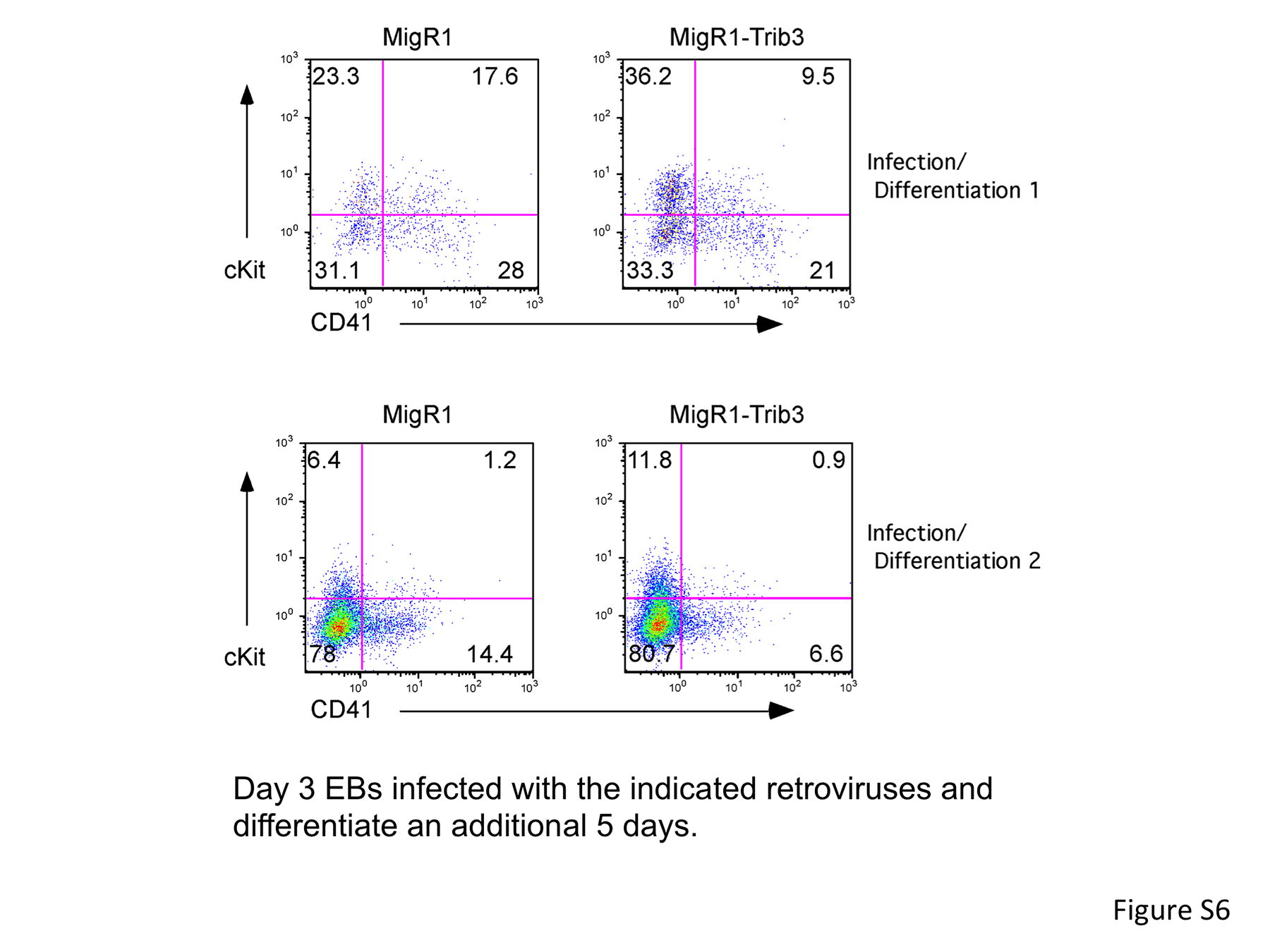

Supplement: S6 Fig — A) Single cell suspensions were prepared from d3 EB cells and infected with MigR1 control or MigR1-Trib3 retrovirus. EBs were reformed by hanging drop, and cultured an additional 5 days. Contribution of the infected (GFP+) cells to the HPC population CD41+, and CD41+cKit+ was evaluated by flow cytometry. Results from 2 independent infections/ differentiations are shown. (TIF) [file pgen.1004959.s006.tif]

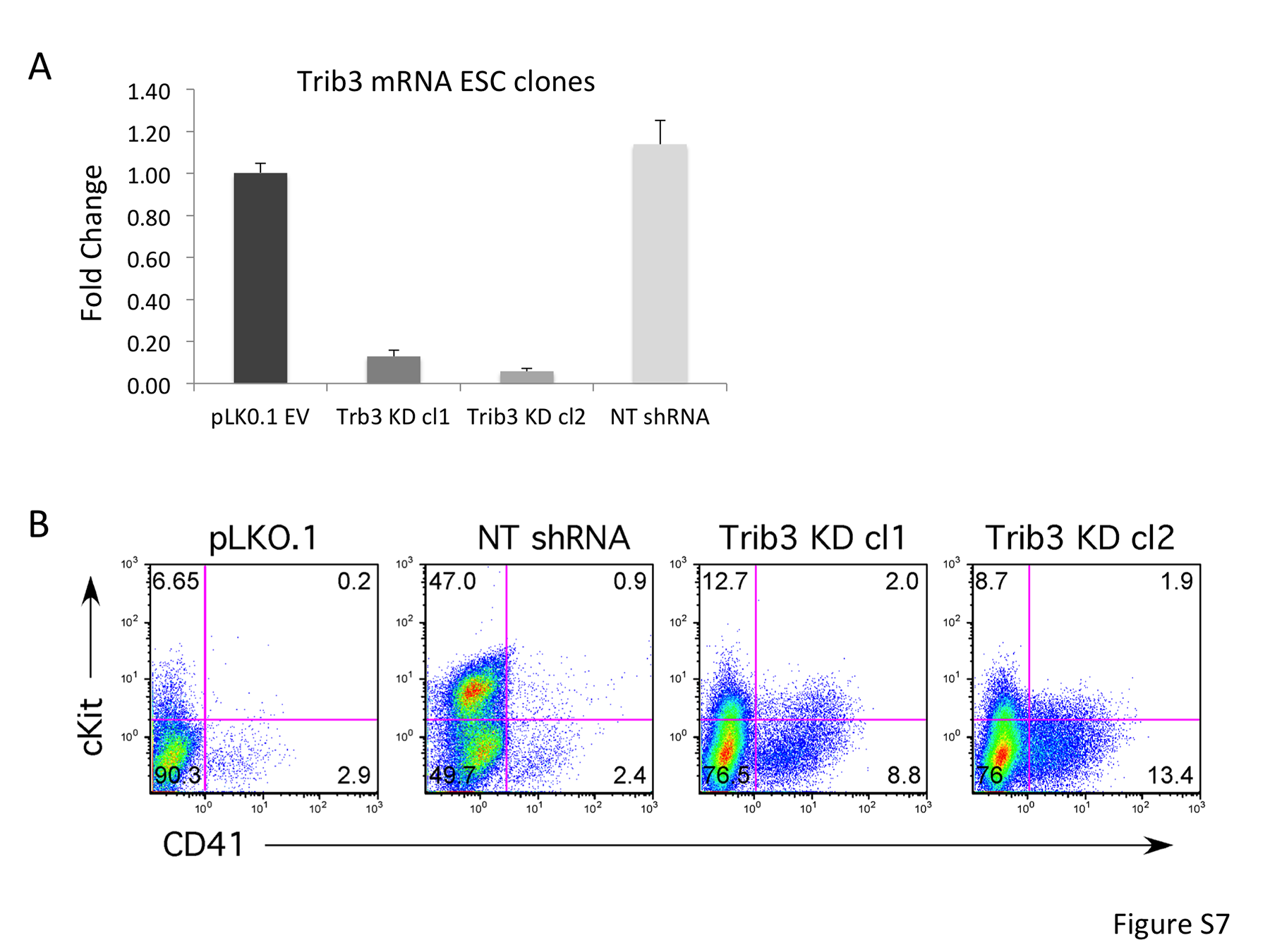

Supplement: S7 Fig — RW4 ESCs were infected with empty vector (pLKO.1), Trib3 shRNA (KD), or non-targeting shRNA expressing lentiviruses. Infected cell clones were generated by selection in puromycin. 1 pLKO.1, 2 independent Trib3 shRNA, and 1 non-targeting (NT) clones were examined. A) Quantitative RT-PCR assaying Trib3 expression in the isolated clones. B) Flow cytometry analysis of CD41 and cKit (CD117) cell surface expression on single cells isolated from EBs generated from the indicated ESC clones. CD41+cKit- population contains primitive HPCs and CD41+cKit+ population contains primitive and definitive HPCs. (TIF) [file pgen.1004959.s007.tif]
